# TRIM4 is associated with neural tube defects based on genome-wide DNA methylation analysis

**DOI:** 10.1186/s13148-018-0603-z

**Published:** 2019-02-01

**Authors:** Henan Zhang, Yi Guo, Hui Gu, Xiaowei Wei, Wei Ma, Dan Liu, Kun Yu, Wenting Luo, Ling Ma, Yusi Liu, Jia Xue, Jieting Huang, Yanfu Wang, Shanshan Jia, Naixuan Dong, Hongyan Wang, Zhengwei Yuan

**Affiliations:** 1 Key Laboratory of Health Ministry for Congenital Malformation, Shengjing Hospital, China Medical University, Shenyang, People’s Republic of China; 2 Obstetrics and Gynecology Hospital, Key Lab of Reproduction Regulation of NPFPC in SIPPR, Institute of Reproduction and Development, Fudan University, Shanghai, People’s Republic of China

**Keywords:** Neural tube defects, Methylation, TRIM4

## Abstract

**Background:**

Neural tube defects (NTDs) are complex abnormalities associated with gene-environment interactions. The underlying cause has not been determined.

**Methods:**

Spinal cord tissues from cases with NTDs and healthy controls were collected. Methylation patterns between cases and normal individuals were compared using 450K Infinium Methylation BeadChip Illumina. DNA methylation analysis by pyrosequencing (PyroMark Q96) and mRNA and protein expression were analyzed using real-time quantitative PCR and Western blotting, respectively. Next-generation and Sanger sequencing were used to determine genetic variants in the target genes.

**Results:**

Spinal cord tissues from cases with NTDs had more hypomethylated than hypermethylated genes. Further evaluation showed that the exon 1 region of TRIM4 was hypomethylated, and TRIM4 mRNA and protein levels were significantly increased in NTDs compared to controls. A rare missense variant (rs76665876) in TRIM4 was found in 3 of the 14 NTD cases but was not related to TRIM4 expression. TRIM4 mRNA levels were significantly increased in cases with hypomethylation and without the rs76665876 variant.

**Conclusion:**

These findings suggest that spinal cord tissues in cases with NTDs had a different genome-wide methylation pattern compared to controls. Abnormal methylation patterns in TRIM4 in immunity pathways might be involved in NTD pathogenesis. Genetic variants in TRIM4 genes only slightly contribute to the etiology of human NTDs.

**Electronic supplementary material:**

The online version of this article (10.1186/s13148-018-0603-z) contains supplementary material, which is available to authorized users.

## Background

Neural tube defects (NTDs) are severe congenital defects in the central nervous system (CNS) that develop during embryogenesis and are due to the failure of neural tube closure, which can lead to neuroepithelium exposure to the environment and consequent neuronal degeneration and impairment [1, 2]. The most common types of human NTDs are spina bifida (myelomeningocele), which is more prevalent, and anencephaly [3]. NTDs affect approximately 300,000 live births annually worldwide, and the incidence in different areas ranges from 0.3 to 199.4 NTDs per 10,000 births [4]. Current NTD incidence in the United States (US) is around 3–6.3 per 10,000 live births every year [4, 5]. The estimated costs of hospital care for a child born with spina bifida range from $21,900 to $1,350,700 in the first year [6]. In China, the incidence of NTDs is 27.4 per 10,000 live births [7].

Both genetic and non-genetic factors participate in the etiology of NTDs. Evidence has supported a multifactorial polygenic model in the genetic pattern of NTDs [8], and many studies of NTD genetics have focused on several candidate genes [9, 10]. Although it has been well demonstrated that periconceptional supplementation of folic acid significantly reduced NTD risk by 50–75% [11], the known risk factors might only account for a small fraction of NTD cases [12]. Numerous epidemiologic studies have suggested that many other non-genetic or environmental factors contribute to NTD risk, including socioeconomic status [13], maternal and paternal ages [14], hyperthermia during early pregnancy [15], maternal medications [16, 17], and toxic heavy metal [18]. However, the etiologies and mechanisms underlying NTD development have yet to be elucidated.

Research has shown that aberrant methylation could play a causal role in increasing the risk of failed neural tube closure [19, 20]. A previous study demonstrated that abnormal global DNA methylation due to the 677C>T variant in the 5,10-methylenetetrahydrofolate reductase gene (MTHFR) significantly increased the risk of human NTDs [21]. Similarly, Wang et al. reported that the methylation levels of long interspersed nucleotide elements were significantly reduced in the brain tissues from NTD samples [22]. Chen et al. reported an association between global DNA hypomethylation and NTD-affected pregnancies in fetal brain tissue [23]. In addition, hypermethylation or hypomethylation of specific genes associated with DNA repair [24], folate receptor [25], imprinting [26, 27], transposon [22], HOX [28], serine/threonine kinases [23], and tight junction pathway [29] has also been shown to play vital roles in NTD development, primarily based on experiments that used brain tissues from NTD animal models. However, given that spina bifida lesions are mainly located in the spinal cord and DNA methylation patterns have been shown to be tissue-specific, it is more appropriate to study spina bifida using spinal cord tissues. Only one previous study focused on genomic DNA methylation patterns in the spinal cord in NTD but DNA methylation patterns in specific genes was not considered [20]. The underlying mechanisms of NTD development using spinal cord tissue at both the genetic and epigenetic levels are warranted.

In the present study, we explored novel aberrant DNA methylation at the genome-wide level in spinal cord tissues from fetuses with NTDs and further examined expression levels of candidate genes. To the best of our knowledge, this is the first study to analyze an association between the TRIM4 gene and NTDs from both genetic and epigenetic perspectives.

## Materials and methods

### Sample collection

Based on the World Health Organization’s International Classification of Diseases, Tenth Edition (ICD-10), NTDs are classified into 4 types: anencephaly (ICD10: Q00), spina bifida (SB) (ICD10: Q05), encephalocele (ICD10: Q01), and congenital hydrocephalus (CHC) (ICD10:Q03). We obtained spinal cord samples from 14 fetuses with NTDs from Shengjing Hospital (Shenyang city, Liaoning province) in China, including 6 cases with spina bifida, 6 cases with congenital hydrocephalus, and 2 cases with a combination of these two defects. Samples of the defective spinal cord were dissected from the terminated fetuses following prenatal diagnosis of an NTD, which were screened through a population-based congenital defect surveillance program. Normal spinal cords were dissected from terminated fetuses with no congenital malformations in the central nervous system and were matched to cases by sex and gestational week (controls group 1). To mitigate any possible bias, we added a separated control group matched to NTD cases by fetal sex and gestational age for real-time PCR analysis of TRIM4 (controls group 2). Features of the cases and controls are shown in Table 1. There were no significant differences between NTDs and controls regarding gender and gestational week. Medical record reviews were used to collect information on obstetric characteristics. Tissue samples from NTD and healthy control fetuses were collected at pregnancy termination by experienced pathologists and stored at − 80 °C until analysis. This study was approved by the Medical Ethics Committee of Shengjing Hospital, China Medical University (2015PS264K). All consent was obtained to use the samples for testing.

**Table 1:** Summary of clinical features and demographic information of samples in this study

NTDs group	Sex	Gestational weeks	Types of deformity	Control group 1	Sex	Gestational weeks	Causes of odinopoeia	Control group 2	Sex	Gestational weeks	Causes of odinopoeia
S1(ES3)	F	29 weeks + 3 days	Spina bifida	N1 (807416)	F	28 weeks + 6 days	Inevitable abortion	S156	F	28 weeks + 1 day	Dead fetus in the uterus
S2(ES6)	F	30 weeks	Spina bifida	N2 (707666)	F	30 weeks + 3 days	Mild pericardial effusion	S104	F	29 weeks	Increased cardiothoracic ratio
S5(ES7)	M	33 weeks	Spina bifida	S90	M	34 weeks	Unplanned pregnancy	N2-5-YANG	M	34 weeks	Dead fetus in the uterus
ES1	F	26 weeks	Meningomyelocele	S108	F	25 weeks + 6 days	Inevitable abortion	S146	F	25 weeks + 3 days	Dead fetus in the uterus
ES2	M	35 weeks + 3 days	Hydrocephaly	S89	M	34 weeks + 5 days	Dead fetus in the uterus	S92	M	34 weeks + 1 day	Unplanned pregnancy
ES4	F	27 weeks + 6 days	Spina bifida	S189	F	26 weeks + 6 days	Inevitable abortion	N2-2-SUN	F	27 weeks + 1 day	Adenoma in the right lung
ES8	F	30 weeks + 6 days	Spina bifida, hydrocephaly	S86	F	32 weeks + 1 day	Unplanned pregnancy	S57	F	32 weeks	Hydrops.
ES9	M	24 weeks	Spina bifida	S158	M	24 weeks + 1 day	Intrauterine distress	S121	M	23 weeks + 2 days	Unplanned pregnancy
NJS2	M	26 weeks + 6 days	Hydrocephaly	S169	M	26 weeks + 4 days	Unplanned pregnancy	S117	M	26 weeks + 3 days	Inevitable abortion
NJS3	F	29 weeks + 6 days	Hydrocephaly	N3	F	31 weeks + 4 days	Biparietal diameter and femur length discrepancy	S148	F	29 weeks	Hydronephrosis in double kidney
NJS4	M	37 weeks + 2 days	Hydrocephaly	N5	M	38 weeks + 2 days	Pleural effusion	N2-8-XIA	M	37 weeks + 2 days	Hydrops.
NJS5	M	29 weeks + 4 days	Hydrocephaly	S168	M	29 weeks + 4 days	Threatened premature labor	N2-10-XU	M	30 weeks	Inevitable abortion
NJS6	M	31 weeks + 1 day	Hydrocephaly	N4	M	30 weeks + 1 day	Eclampsia	S83	M	30 week + 3 days	Pleural effusions
NJS8	F	24 weeks + 1 day	Spina bifida, hydrocephaly	S109	F	25 weeks + 6 days	Inevitable abortion	N2-4-WANG	F	24 weeks	Unplanned pregnancy

### DNA extraction and bisulfite conversion of genomic DNA

Genomic DNA was extracted from spinal cord tissues with 0.5% sodium dodecyl sulfate lysis buffer and protease K (1.5 mg/mL) for nuclear protein digestion at 56 °C for 1 h. Total genomic DNA was harvested using a QIAamp DNeasy Blood and Tissue Kit (QIAGEN, Hilden, Germany), followed by precipitation with 70% alcohol. For each sample, genomic DNA was quantified by NanoDrop ND-1000, and 500 ng genomic DNA was used for bisulfite conversion using an EpiTect Whole Bisulfitome Kit (Qiagen). Bisulfite-converted genomic DNA was eluted and stored at − 20 °C until use.

### DNA methylation analysis

DNA from spinal cord tissues from the NTD group (three cases) and control group (three cases, matched by sex and gestational week) were used for the methylation array assay. Genomic DNA was extracted, purified, and bisulfite converted. Samples were randomized across 3MSA-4 plates for processing based on instructions for the Illumina Infinium HumanMethylation450 BeadChip [30]. To minimize batch effects, cases and controls were randomly distributed into different arrays. Raw intensity was read using Illumina GenomeStudio Software 2011.1, and background normalization was performed.

The raw data was first pretreated with R software minfi package. R software Illumina Methylation Analyzer (IMA) was then used to screen methylation levels and regions in the samples [31, 32]. Beta value (*β* value) was used as an indicator of methylation for each locus in each sample (*β* values range from 0 to 1, corresponding to completely unmethylated and fully methylated sites, respectively). Delta beta (Δ*β*) is defined as the difference in *β* values between the two groups in which the greater absolute value represents a greater degree of difference. DiffScore is the parameter and model measuring differences as provided by the Illumina Company in which a greater absolute value indicates a more significant difference. Delta beta and DiffScores were both used to differentially screen for methylated genes. Detection *p* values represent the confidence levels of chip signal values. Detection probes with a *p* value > 0.05 were not reliable and were therefore excluded from further analysis.

### Differential methylation gene analysis

To identify differentially methylated genes between NTD cases and controls, the following five filters were applied: (i) absolute *β* value difference > 0.10, (ii) DiffScore > 13, (iii) detection *p* value < 0.05, (iv) participate and play an important role in multiple pathways, and (v) differentially methylated CpGs located in the transcriptional regulatory region of the gene, such as 5′ untranslated region (UTR), transcriptional start sites (TSS) 200, first exon, 3′UTR, and more.

To identify enriched gene functions associated with differentially methylated regions (DMRs), Gene Ontology (GO) analysis was performed using online gene enrichment tools from Shanghai Biotechnology Corporation (http://enrich.shbio.com/index/ga.asp). The Fisher accuracy test was used for enrichment analysis with the clusterProfiler package [33], which was released within the Bioconductor project. Genes associated with 450K CpG sites were ranked by unadjusted *p* values (from smallest to largest) and computed hypergeometric *p* values for overrepresentation of each biological process Gene Ontology (GO) category. A term containing 10 or more genes as well as a *p* value < 0.05 was considered a selection criterion for interested terms.

### Real-time quantitative PCR

Total mRNA from spinal cord tissues from 14 cases and 28 controls (including 3 samples in microarray analysis) was extracted with TRIZOL (Invitrogen, Carlsbad, CA, USA) according to the manufacturer’s instructions. Complementary DNA (cDNA) was synthesized using a PrimeScript™ RT reagent kit (TAKARA, Japan) with mRNA as the template. Transcript expression of candidate genes was evaluated using specific primers in a 20 μL reaction (2 μL of template cDNA, 1 μL of 10 μmol/L each primer, 10 μL of 2× SYBR Green Master Mix, 0.4 μL of ROXII, and 5.6 μL of ddH2O) on an ABI Prism 7500HT sequence detection system (Applied Biosystems) with the following cycling parameters: predenaturation at 95 °C for 30 s, 45 cycles of denaturation at 95 °C for 5 s, and annealing at 60 °C for 20 s. Relative levels of target gene expression were calculated according to the ΔΔCt method [34] and normalized to glyceraldehyde 3-phosphate dehydrogenase (GAPDH) gene expression. The primers for candidate genes and GAPDH are shown in Additional file 1: Table S1.

### Pyrosequencing and sequence analysis

DNA from spinal cord tissues obtained from 14 cases and 14 controls (including 3 samples in microarray analysis) was used for pyrosequencing and sequence analysis. Pyrosequencing was performed using the PSQ Gold SQA reagent kit (Qiagen) on a Pyromark Q96 ID platform (Qiagen, Germany) according to the manufacturer’s instructions. Briefly, 50 μL biotinylated PCR products were mixed with 3 μL streptavidin sepharose beads (Amersham Biosciences AB, Sweden) and 47 μL binding buffer, followed by shaking at 2000 rpm for 10 min. The immobilized complex was captured by using a vacuum prep tool. To dissociate and discard the unbiotinylated strand, single strand purification was performed. The beads that bound to single biotinylated strands were released to a 96-well microtiter plate to which 49 μL annealing buffer, and 1 μL complementary sequencing primer (pBR-V1.AS) had been added. The processed mixture was loaded onto the PyroMark ID system for pyrosequencing set with 10 cycles of ATCG dispenses. The obtained sequences were directly used to search the database constructed. Results were identified as organisms with a 100% DNA sequence matching. Sequences for pyrosequencing primers are shown in Additional file 2: Table S2.

### Next-generation sequencing and Sanger sequencing

Genomic structures of human TRIM4 genes were confirmed by NCBI GenBank (NM_033091.2 and NM_033091.3). Gene variants in TRIM4 were detected by next-generation sequencing. The Agilent SureSelect XT Custom enrichment system was used to construct genomic DNA-fragment libraries and target enrichment of TRIM4. Each fragmented genomic DNA library was connected to an index adapter, and the connected libraries were gel purified and PCR amplified (Phusion, Thermo Scientific). The Agilent 2100 biologic analyzer was used to determine the quantity and quality of the library. Forty-eight libraries were pooled in total and then hybridized to RNA library baits, after which the targeted sequences were purified and amplified (Herculase II fusion, Stratagene). Sequencing was performed on an Illumina HiSeq2000 DNA sequencer (version 3). Sequence alignment was performed with BWA software (Li and Durbin, 2009) based on the h19 database.

To confirm genotyping results for TRIM4 from next-generation sequencing, Sanger sequencing was designed to detect variants in all exons, 1 kb upstream of the transcription start site, and 3′UTRs in TRIM4. The primer design tool (https://www.ncbi.nlm.nih.gov/tools/primer-blast/) was used to design specific primers. Extracted DNA was amplified by polymerase chain reaction (PCR) using P*fu* DNA Polymerase, MgCl_2_ (25 mM), 10× PCR buffer (without Mg^2+^:100 mM Tris-HCl (pH 8.8, 25 °C), 500 mM KCl, 0.8% (*v*/*v*) Nonidet P40), and dNTP (10 mM) (Sangon Biotech, Shanghai, China). The optimal annealing temperature for PCR was 58 °C. Amplified PCR products were purified and recollected using a SanPrep Column DNA Gel Extraction Kit (Sangon Biotech, Shanghai, China). DNA sequencing was performed on an ABI 3730xl DNA sequencer (Applied Biosystem, Foster City, USA) following the manufacturer’s standard sequencing protocols. For data analysis, the dbSNP in NCBI, Genome 1000, and ExAC database was used as a reference dataset for rare variant allele frequency in a Chinese Han population.

### Western blotting

Spinal cord tissues from 14 cases and 14 controls, including 3 samples in microarray analysis, were lysed in ice-cold RIPA buffer (Solarbio, R0010, China) with 1 mM phenylmethanesulfonyl fluoride (PMSF). After quantification using the BCA method, proteins were electrophoresed on 10% SDS-polyacrylamide gels and transferred to polyvinylidene difluoride (PVDF) membrane (Millipore, USA). The membranes were blocked with 5% non-fat milk in PBST containing 0.1% Tween-20 for 60 min and incubated with primary rabbit anti-RNF87 (TRIM4) (ab26300, 1:800, Abcam, Cambridge, UK) and a mouse anti-GAPDH antibody (sc-365062, 1:10000, Santa Cruz Biotechnology) overnight at 4 °C, followed by incubation with secondary polyclonal goat anti-rabbit HRP-conjugated (ZDR-5306, 1:2000, ZSGB-bio, Beijing, China) or polyclonal goat anti-mouse HRP-conjugated antibody (ZDR-5307, 1:2000, ZSGB-bio, Beijing, China) for 2 h at room temperature. Signals were visualized using enhanced chemiluminescence (ECL, Millipore, USA) reagents. Optical density values of each protein divided by the loading control (GAPDH) were regarded as the relative density of each protein.

### MTHFR and MTRR polymorphism analysis

Polymorphism analysis was assessed at three loci in each case: at nucleotide 677C>T (rs1801133) and 1298A>C (rs1801131) in MTHFR and 66A>G (rs1801394) in methionine synthase reductase (MTRR). Human MTHFR and MTRR gene polymorphism detection kit (YZYMT-014, Wuhan, China) was used to determine the genotype of the sample by comparing changes in Ct values of wild-type and mutant primers by a real-time fluorescent TaqMan probe PCR method. PCR was performed on an ABI Prism 7500HT sequence detection system (Applied Biosystems) with the following cycling parameters: 42 °C for 5 min, 94 °C for 3 min, 45 cycles of denaturation at 94 °C for 15 s, and annealing at 60 °C for 60 s.

### Statistical analysis

The genotype and alleles of MTHFR and MTRR were computed by direct counting. Alleles and genotypes distribution of MTHFR and MTRR were evaluated by Fisher’s exact test using the SPSS software package version 20.0. A non-parametric test was used to compare the differences in methylation and expression levels between NTDs and controls using GraphPad Prism 6 (GraphPad Software, California, USA). All data are represented as the mean ± standard error of the mean (SEM). *p* value less than 0.05 was considered statistically significant.

## Results

### Screening for differentially methylated genes

To investigate whether DNA methylation was different between controls and NTD cases, we measured global DNA methylation in spinal cord tissues using the Illumina Infinium Human Methylation450 BeadChip, which examines over 485,000 CpG sites spanning the whole human genome.

**Fig. 1:**
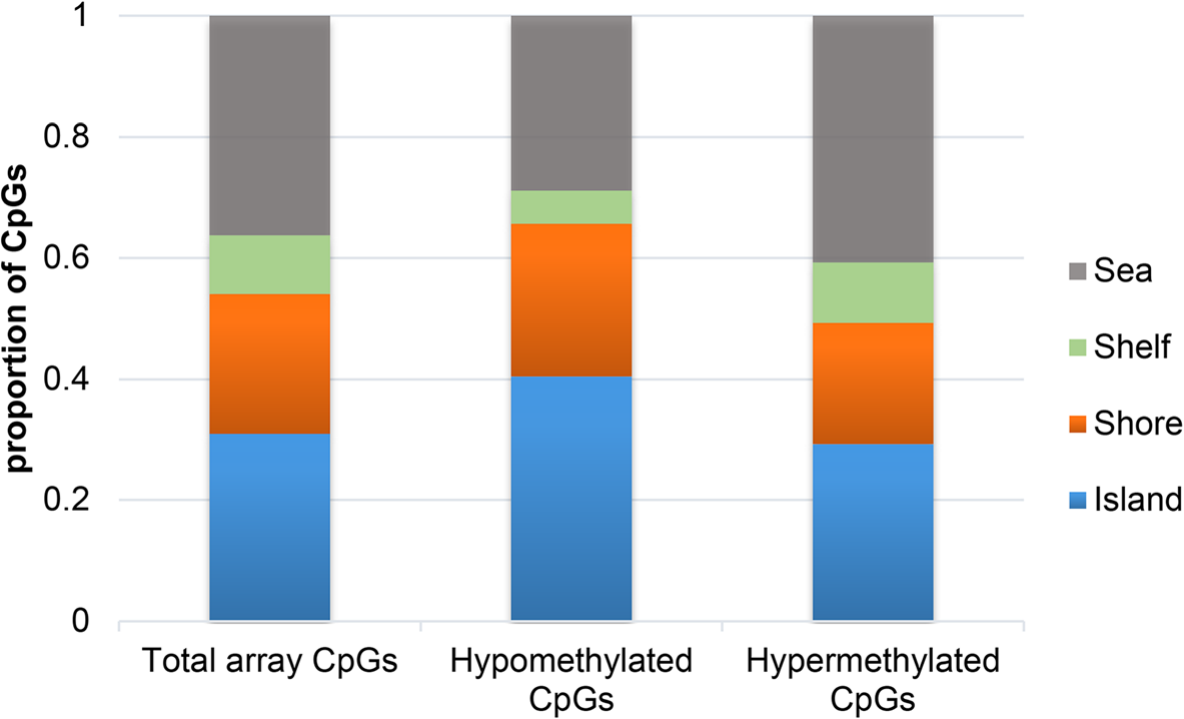
Percentage of CpGs at different sites in samples from NTD spinal cord tissue. Both hypomethylated and hypermethylated DMSs were preferentially located in CpG islands rather than shores and shelves

**Fig. 2:**
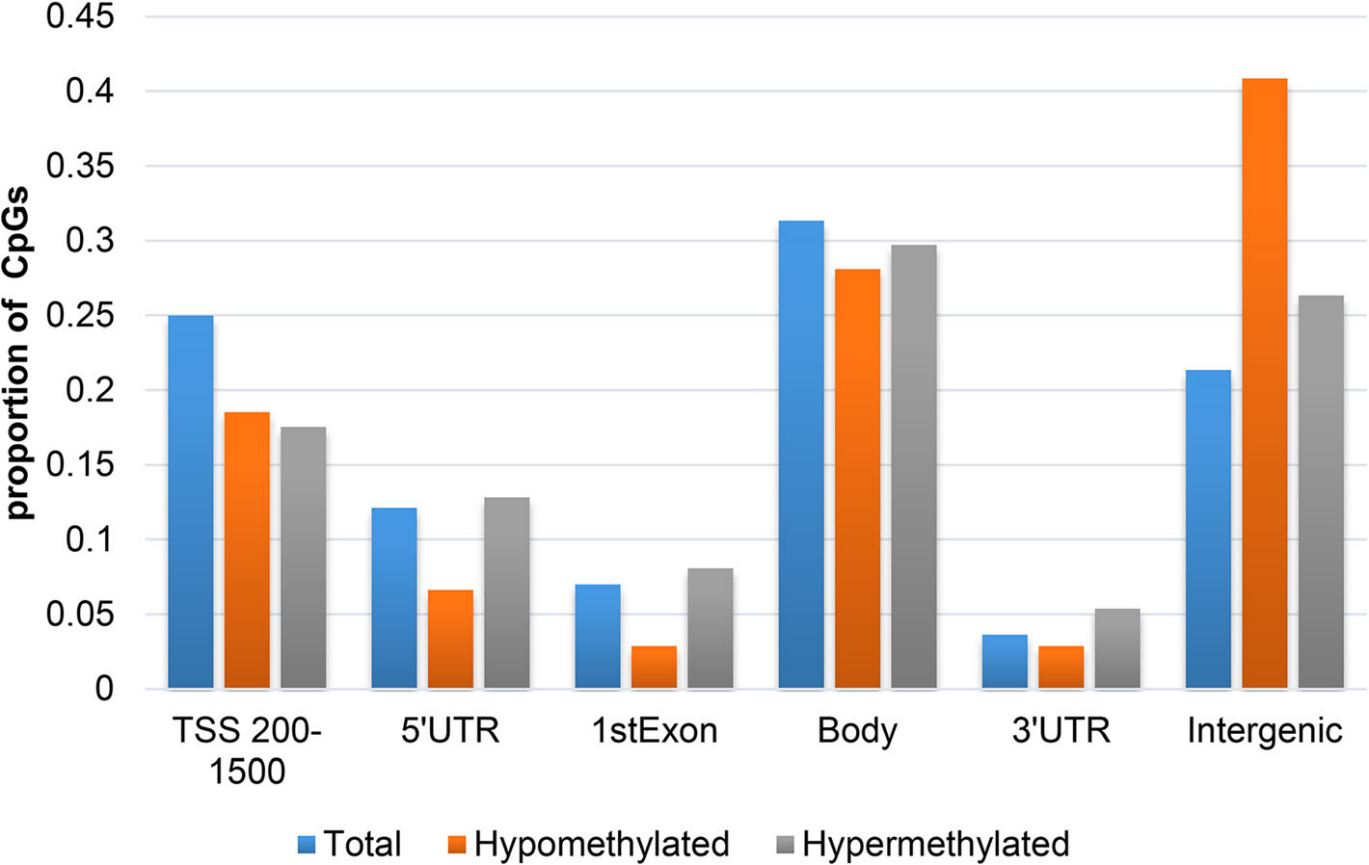
Overall distribution of DMRs relative to RefSeq genes. A significant enrichment in hypomethylated CpGs in intergenic regions is shown. Hypomethylated and hypermethylated DMSs were referentially distributed within the TSS200-TSS1500 region

Filtered by absolute Δ*β* difference > 0.10 and DiffScore > 13, a total of 4648 differentially methylated sites (DMSs) were screened out, of which 1987 sites were hypermethylated and 2661 were hypomethylated. After filtering out unreliable chip signal values (detection *p* value > 0.05), 461 DMSs were identified, including 330 hypomethylated sites and 131 hypermethylated sites. These DMSs were distributed in 243 genes, including 156 hypomethylated genes, 83 hypermethylated genes, and 4 genes with both hypermethylated and hypomethylated sites (ACTR3C, GALNT9, HLA-DQB1, HLA-DQB2). There were more hypomethylated sites in HLA-DQB1 and HLA-DQB2 genes than hypermethylated sites. There were more hypermethylated sites in the GALNT9 gene than hypomethylated sites. The number of hypomethylated and hypermethylated sites in the ACTR3C gene was the same. CpG distribution of DMSs was analyzed and compared to CpG distribution in the Illumina 450K array, which covers 99% of annotated RefSeq genes and exhibits a wide distribution of probes among CpG islands, shores (2 kb flanking the islands), shelves (2 kb flanking the shores), and sea (regions outside the previous three categories). The results showed that both hypomethylated and hypermethylated DMSs in NTD spinal cord tissues were preferentially situated in CpG islands rather than shores and shelves (Fig. 1). The distribution of DMSs in the gene region was also analyzed and compared to RefSeq genes. Interestingly, the global distribution of DMSs relative to RefSeq genes showed a significant enrichment in hypomethylated CpGs in the intergenic regions. In mainly translating regions, including TSS200-TSS1500, 5′UTR, exon 1, and 3′UTR, hypomethylated and hypermethylated DMSs were preferentially distributed in the TSS200-TSS1500 region (Fig. 2).

### Bioinformatics analysis of candidate genes of interest

We performed Gene Ontology analysis of 243 methylated differential genes, of which only 131 hypomethylated and 78 hypermethylated genes were annotated in GO databases. After sorting the 131 hypomethylated genes according to the *p* value, we found 7 terms with more than 10 gene assemblies in biological process (BP) GO terms. They were GO:0001816, cytokine production (7.63%); GO:0045087, innate immune response (11.45%); GO:0002520, immune system development (8.40%); GO:0050776, regulation of immune response (9.92%); GO:0006955, immune response (14.5%); GO:0006952, defense response (15.27%); and GO:0010033, response to organic substance (21.37%) (Additional file 3: Table S3). Hypermethylated genes were detected in categories associated with lipid, cellular lipid, and organic acid metabolic processes; establishment of localization in the cell; cellular localization; response to organic substances; protein localization; transport; and transmembrane transport (Additional file 4: Table S4). A further selection of target genes with a significant number of DMSs in a transcriptional regulation domain identified 5 immune specific hypomethylated genes (TLR1, TRIM4, MAP2K2, CALCOCO2, GNAS) and 5 hypermethylated genes (SMPD3, EGFR, HSPB7, MAGT1, SCT) involved in metabolic processes, localization, and transportation pathways.

### Candidate gene expression level analysis

**Fig. 3:**
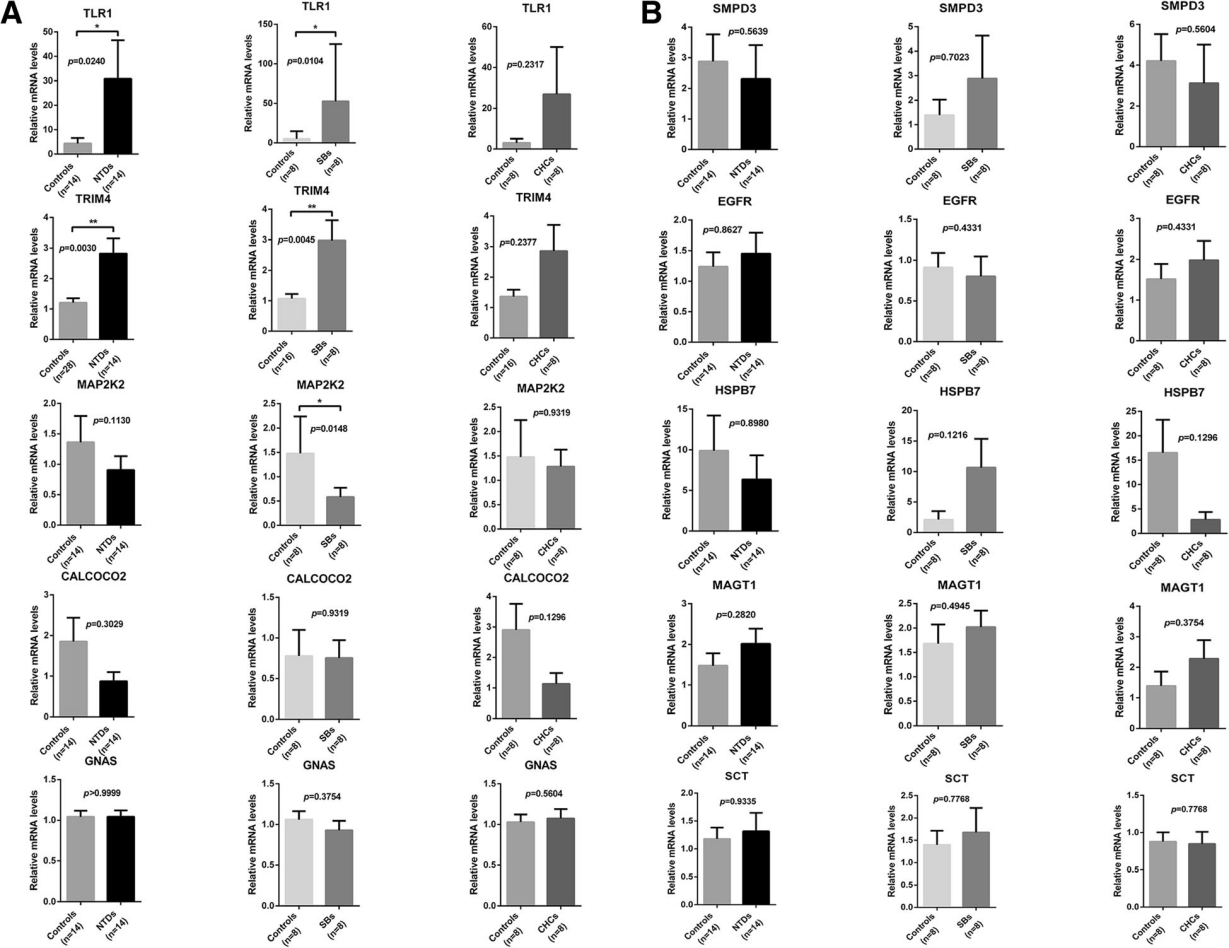
mRNA expression analysis of differential methylation genes using real-time PCR. **a** Comparison of hypomethylated DMG expression between NTDs and controls, SBs and controls, and HCs and controls, respectively. **b** Comparison of hypermethylated DMG expression between NTDs and controls, SBs and controls, and HCs and controls, respectively. Data are represented as the mean ± SEM. *p < 0.05, **p < 0.01

To reveal the relationship between methylation status and gene expression levels of these target genes, real-time quantitative PCR was performed to detect mRNA expression levels. Of the hypomethylated differentially methylated genes (DMGs), TRIM4 and TLR1 expression was higher in NTDs, while MAP2K2, CALCOCO2, and GNAS expression showed no significant difference (Fig. 3a). No significant differences in expression levels of hypermethylated DMGs were found in NTDs (Fig. 3b). When evaluating NTD subgroups, we found that TRIM4 and TLR1 expression was increased, but MAP2K2 expression was decreased in cases with SB compared to controls. There were no significant differences in TRIM4, TLR1, and MAP2K2 expression in cases with CHC (Fig. 3a). No significant differences in expression levels of hypermethylated DMGs were found in both cases with SBs and CHCs (Fig. 3b). To mitigate any possible bias, we added a separated control group (control group 2) matched to NTD cases by fetal sex and gestational age for real-time PCR analysis of TRIM4. In line with the results of control group1, the expression of TRIM4 was significantly higher in the NTD group compared to controls group 2. Since the results of the two control groups were consistent, we combined the two control groups into one group (Fig. 3a).

**Fig. 4:**
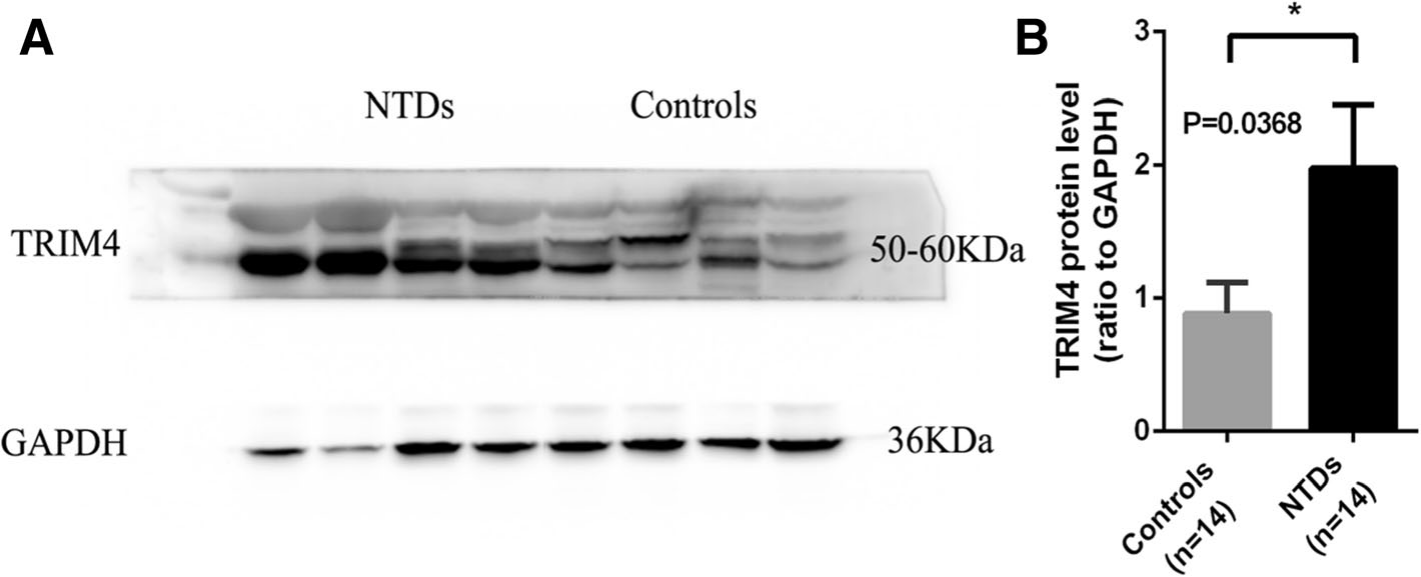
Western blot analysis of TRIM4 expression in NTDs and controls. **a** Representative image of TRIM4 expression in different individuals. **b** Quantification of TRIM4 protein levels in NTDs (black bar) and controls (gray bar). Data are represented as the mean ± SEM. *p < 0.05 vs. controls

Western blot analysis was performed to further verify changes in TRIM4 protein expression levels in NTDs. In accordance with the results obtained from real-time PCR, we found that TRIM4 protein levels were significantly increased in NTDs compared with controls (Fig. 4).

### Validation of methylation levels in differentially expressed genes by pyrosequencing

**Fig. 5:**
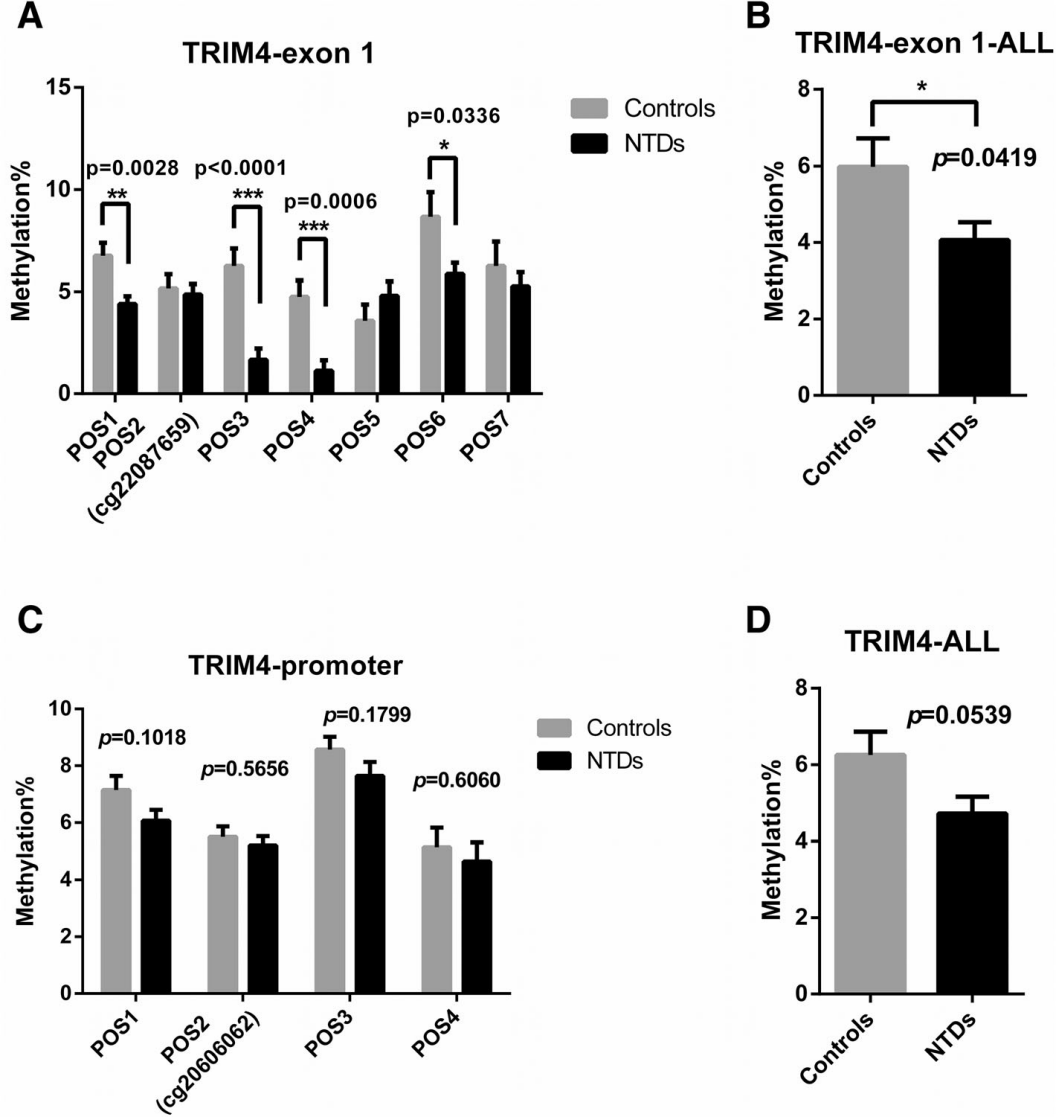
Pyrosequencing to identify methylated CpG sites in TRIM4. DNA methylation levels at each CpG site (POS) by percentage were analyzed by pyrosequencing in NTDs (black bar) and controls (gray bar) in exon 1 (**a**, **b**), promoter region (**c**), and both regions (**d**). Data are represented as the mean ± SEM. *p < 0.05, **p < 0.01, ***p < 0.001 vs. controls

**Fig. 6:**
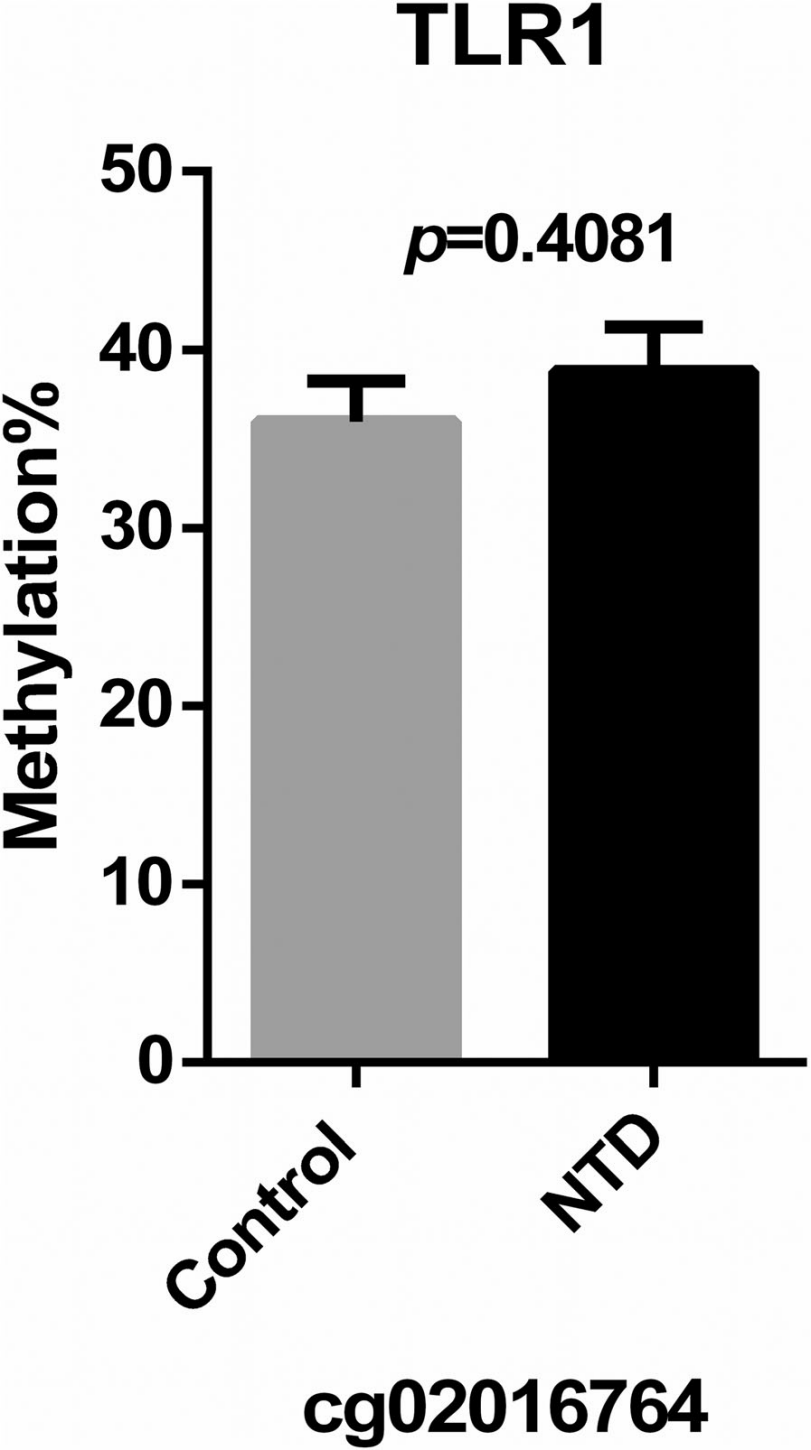
Pyrosequencing to identify methylated CpG sites in TLR1. DNA methylation levels at each CpG site (POS) by percentage were analyzed by pyrosequencing in NTDs (black bar) and controls (gray bar) in cg02016764. No significant difference was found between NTDs and controls

Pyrosequencing was used to identify methylation levels in different transcription regulatory regions in TRIM4, TLR1, and MAP2K2 from 14 NTD samples and matched controls. However, pyrosequencing was only conducted for TRIM4 and TLR1 due to the low primer score for MAP2K2. CpG site information for pyrosequencing is shown in Additional file 5: S1. The regions targeted for pyrosequencing contain differential methylation sites obtained from microarray analysis as well as surrounding CG sites. We analyzed seven CpG sites in exon 1 of TRIM4 and found that the degree of methylation in four sites (POS1, POS3, POS4, POS6) was significantly decreased in NTDs compared with controls (Fig. 5a). Total methylation of the seven CpG sites in exon 1 between NTDs and controls was also significantly different (NTDs: mean ± SEM = 4.057 ± 0.4712, *N* = 14; controls: mean ± SEM = 5.977 ± 0.7482, *N* = 13. *p* = 0.0419) (Fig. 5b). None of the four CpG sites in the promoter region showed differences in methylation levels between NTDs and controls (Fig. 5c). Total methylation levels in these two regions showed no significant difference (NTDs: mean ± SEM = 4.656 ± 0.3877, *N* = 14; controls: mean ± SEM = 5.971 ± 0.6334, *N* = 13, *p* = 0.0539) (Fig. 5d). These results indicate that the exon 1 region in TRIM4 shows significant hypomethylation. The sequence information of the regions targeted for pyrosequencing in TRIM4 was shown in Additional file 6: S2. Pyrosequencing results of the TLR1 gene showed no difference in methylation levels between NTDs and controls (Fig. 6).

### TRIM4 mutation analysis

To investigate whether SNPs contribute to increased TRIM4 gene expression in NTD cases, we reanalyzed the previous genomic DNA sequencing results obtained from a different population of 100 NTD cases that was independent of the 14 NTD fetus samples. Forty rare variants (MAF < 0.01) were identified, including 4 upstream variants, 32 intron variants, 1 downstream variant, 1 3′UTR variant, 1 missense variant, and 1 synonymous variant. We also detected an exon region around the DMS in the 14 spinal cord tissues and found 3 3′UTR variants, 3 missense variants, 2 synonymous variants, 1 stop-gain variant, and 6 novel variants. Of these, 3 3′UTR variants (rs2572009, rs1048705, rs2572010), 1 missense variant (rs76665876) and 2 synonymous variants (rs2247761, rs2247762) were identified in both sequencing methods. Only the missense variant (rs76665876) is rare and was predicted to have a potential pathogenic possibility by using Polyphen and AvSIFT programs (Polyphen score, 0.980; SIFT score, 0.14). Therefore, we also analyzed the association between mRNA expression and the rare missense variant (rs76665876) in the 14 spinal cord samples with NTDs. The rs76665876 variant was found in 3 of the 14 NTD cases, and TRIM4 gene expression was not significantly different. There were 8 cases with hypomethylation without the rs76665876 variant in NTDs, and TRIM4 mRNA expression was significantly increased in these cases. Collectively, the mRNA expression, DNA methylation, and sequencing data suggest that genetic variants in TRIM4 genes only slightly contribute to the pathogenesis of human NTD. The main factor affecting TRIM4 mRNA levels in NTDs is the changes in DNA methylation.

### MTHFR and MTRR polymorphism analysis

Three SNPs (MTHFR C677T, A1298C, and MTRR A66G) that were previously identified as risk factors for NTD offspring were evaluated in the samples we collected. For the 677C>T and 1298A>C polymorphisms in MTHFR, there was no statistically significant difference in genotype distribution (*p* values of 0.821 and 0.385, respectively) and allele frequency (*p* values of 0.785 and 0.422, respectively) between NTDs and controls. Similarly, no significant differences were observed in genotype distribution (*p* = 0.214) and allele frequency (*p* = 1.000) for 66A>G in MTRR between NTDs and controls (Table 2). To confirm the MTHFR and MTRR variants in spinal cord samples, we detected the same SNPs in skin tissue from the 14 NTD fetuses. The same results were found in both tissue types.

**Table 2:** Alleles and genotypes distribution of MTHFR and MTRR

	Genotype/allele	Controls, *n* (%)	NTDs, *n* (%)	*p*	OR (CI 95%)
Sample size (*n*)		14 (100)	14 (100)		
MTHFR C677T					
Genotype frequency	CC	1 (7.1)	3 (21.4)	0.643	
	CT	8 (57.1)	6 (42.9)		
	TT	5 (35.7)	5 (35.7)		
Allele frequency	C	10 (35.7)	12 (42.9)	0.785	0.741 (0.253–2.173)
	T	18 (64.3)	16 (57.1)		
MTHFR A1298C					
Genotype frequency	AA	9 (64.3)	12 (85.7)	0.385	
	AC	5 (35.7)	2 (14.3)		
	CC	0 (0)	0 (0)		
Allele frequency	A	23 (82.1)	26 (92.9)	0.422	0.354 (0.063–2.002)
	C	5 (17.9)	2 (7.1)		
MTRR A66G					
Genotype frequency	AA	5 (35.7)	7 (50.0)	0.214	
	AG	9 (64.3)	5 (35.7)		
	GG	0 (0)	2 (14.3)		
Allele frequency	A	19 (67.9)	19 (67.9)	1.000	1.000 (0.326–3.070)
	G	9 (32.1)	9 (32.1)		

To investigate whether the variants in MTHFR (C677T and A1298C) and MTRR (A66G) were associated with methylation levels of TRIM4, we compared TRIM4 methylation levels between NTDs with and without homozygote mutants in MTHFR and/or MTRR. No difference in methylation levels was found between the two groups (NTDs with homozygote mutants in MTHFR and/or MTRR: mean ± SEM = 4.617 ± 0.5224, *N* = 6; NTDs without homozygote mutants in MTHFR and/or MTRR: mean ± SEM = 3.638 ± 0.7189, *N* = 8, *p* = 0.3230).

## Discussion

In the present study, we explored differences in genome-wide DNA methylation status in NTD cases compared with healthy controls in a Chinese population. Biological processes were identified in nine and seven differentially hypermethylated and hypomethylated CpG sites, respectively. After pyrosequencing in a larger sample size, we confirmed for the first time that TRIM4 was hypomethylated in NTDs. Additional quantitative real-time PCR and Western blot showed that TRIM4 mRNA and protein expression levels were higher in NTDs compared to controls.

TRIM4 is a member of the tripartite motif (TRIM) family of proteins, and its cellular function has not been identified. Recently, TRIM4 has been shown to associate with RIG-I and regulate the process of K63-linked ubiquitination. Overexpression of TRIM4 potentiated virus-triggered activation of IRF3 and NF-kB, as well as IFN-b induction, whereas knockdown of TRIM4 had opposite effects, suggesting that TRIM4 is an important regulator of virus-induced IFN induction pathways during innate antiviral responses [35]. Mutations in TNIP1, a gene whose main role is downregulation of the NF-kB pathway, were found in NTD patients [36], and knockout of genes involved in the NF-kB pathway, including Bcl10, IKKa, IKKb, and TRAF6, showed NTD-related phenotypes in embryonic stages in mice [37–39]. These results suggest that the genes involved in the NF-kB pathway demonstrate a relationship with NTDs and that TRIM4 might participate in the etiology of NTDs via regulation of innate immune responses, including NF-kB signaling. Another study showed that TRIM4 forms distinct cytoplasmic speckle-like structures that transiently interact with mitochondria to induce mitochondrial aggregation and sensitize cells to H_2_O_2_-induced death [40]. H_2_O_2_ is a major ROS entity generated from mitochondrial respiratory complex I and III during stress conditions, and evidence has demonstrated that TRIM4 sensitizes cells to H_2_O_2_-induced death [41, 42]. Previous evidence showed that mitochondria-related biological processes play a role in the etiology of NTDs [43, 44]. Thus, TRIM4 might also affect NTDs through regulation of mitochondrial function.

In the present study, we found that NTD cases had more hypomethylated than hypermethylated CpG sites, which is in accordance with studies in human placenta and leukocytes reported by Zhang et al. and Rochtus et al., respectively [45, 46]. Previous research [47] in Han Chinese NTD pedigrees showed that genes with hypermethylations clustered in pathways associated with epithelial-to-mesenchymal transition (ZEB2, SMAD6, and CDH23) and folic acid/homocysteine metabolism (MTHFD1L), although significant differences were not detected in our results. This discrepancy might be due to different tissues used in these two studies. In our study, spinal cord tissue from spina bifida cases was used. In addition, Price et al. [20] did not identify different methylation sites in NTD spinal cord tissue. However, the present study recognized several differentially methylated genes related to immunity pathways, as well as metabolism, localization, and transportation processes. These differences might due to different ethnicities evaluated.

MTHFR gene mutations in 677CT (C to T) and 1298 AC (A to C) have been identified to increase heat sensitivity and decrease enzyme activity in MTHFR, resulting in human disorders including neural tube defects [48]. MTRR 66A>G mutation at cDNA nucleotide position 66 converts an isoleucine to a methionine residue at amino acid position 22 (I22M). This MTRR polymorphism may interfere with methionine synthase interaction and could be associated with an increased NTD risk in offspring [49, 50]. MTHFR C677T, A1298C, and MTRR A66G were evaluated in this study. Given the small population size, there was no statistically significant difference in the distribution of genotypes and allele frequencies for 677C>T, 1298A>C polymorphism in MTHFR, and 66A>G polymorphism in MTRR between NTDs and controls, which is consistent with the results reported in Price et al. [20] and van der Linden et al. [51]. To investigate whether the variants in MTHFR (C677T and A1298C) and MTRR (A66G) contribute to methylation levels in TRIM4, we compared the levels in TRIM4 between NTDs with and without homozygote mutants in MTHFR and/or MTRR. No difference in methylation levels was found between the two groups, indicating that these variants had no influence on methylation levels in TRIM4.

Both of the gene variants and epigenetic changes could affect embryonic development by regulating gene expression. Therefore, it is important to analyze both genetic mutations and methylation changes for NTDs simultaneously. The rare missense variant rs76665876 was detected in both sequencing analyses. TRIM4 mRNA levels were not significantly different in cases with the rs76665876 variant but were significantly increased in cases with hypomethylation and without the rs76665876 variant, indicating that altered TRIM4 mRNA levels may be associated with changes in DNA methylation and not related to the rs76665876 variant. The relationship between this variant and NTDs should be further validated in a larger sample size in the future. Our findings suggest that changes in TRIM4 gene expression in NTDs is not likely due to a genetic cause, but an epigenetic consequence.

One of the limitations in this study was a small sample size, but we have more than 95% statistical power to detect the expression and DNA methylation difference at a significance level of 0.05 using a two-tailed test. Meanwhile, we have taken some strategies in the experimental design to mitigate the bias caused by the small sample size. Fetal sex and gestational age may be the confounding factors in the present study. To mitigate the confounding bias, we matched the controls to NTD cases by fetal sex and gestational age. To mitigate the random bias, we added another isolated control group (*n* = 14) matching with NTD cases for real-time PCR analysis of TRIM4. The results of the two control groups were consistent. Another limitation is that although we detected abnormal methylation and aberrant TRIM4 expression, the underlying role of TRIM4 in the etiology of NTDs remains unclear. Therefore, additional studies are needed to validate the observed association of TRIM4 DNA methylation with the risk for NTDs. In addition, it is still unknown whether methylation changes are the result of any parent-of-origin effects. Although all parents in the present study were not affected by NTDs, the possibility of direct parent-child transmission cannot be ruled out. Further studies, including the methylation status in both parents and offspring, should be performed to explore whether abnormal methylation patterns are de novo or inherited.

## Conclusions

Identification of unique methylation patterns in Chinese NTD subjects was the most vital finding in this study. Methylation data was combined with next-generation sequencing approaches to explore NTD etiology. This study identified a new pathogenic mechanism for the contribution of hypomethylation of TRIM4 in immunity pathways to human NTDs. These data suggest the need to focus on immune pathways in exploring the etiology of NTDs in future studies.

## Additional files

Additional file 1: **Table S1.** Primers for real-time PCR. (DOCX 18 kb)

Additional file 2: **Table S2.** Primers for pyrosequencing. (DOCX 17 kb)

Additional file 3: **Table S3.** Functions of genes with differential hypomethylation. (Note: yellow marker represents that there are more than ten genes in this GO). (DOCX 25 kb)

Additional file 4: **Table S4.** Functions of genes with differential hypermethylation. (Note: yellow marker represents that there are more than ten genes in this GO). (DOCX 27 kb)

Additional file 5: S1. CpG site information for pyrosequencing in TRIM4, TLR1, and MAP2K2. Pyrosequencing was performed for identifying the methylation level of different transcription regulatory regions in TRIM4, TLR1, and MAP2K2 in a larger sample size. However, the pyrosequencing test was only conducted in TRIM4 and TLR1 due to the low primer score of MAP2K2. The cg20606062, cg09654046, cg22087659, cg02016764, and cg24748945 mentioned below are the probe number of the differential methylation site in the microarray analysis. (DOCX 29 kb)

Additional file 6: S2. The sequence information of the regions targeted for pyrosequencing in TRIM4. (Notes: the underline represents the pyrosequencing fragment of *TRIM4* in promoter and exon 1. Yellow font represents the primers of Sanger sequencing. Red font represents the detected CpG site. Blue font represents the first exon of TRIM4. Green font represents the missense mutation. Purple highlight represents the cg09654046, cg20606062, and cg22087659, respectively). (DOCX 14 kb)

## Abbreviations

3′UTR: 3′ Untranslated regions
BP: Biological process
cDNA: Complementary DNA
CHC: Congenital hydrocephalus
CNS: Central nervous system
DMGs: Differentially methylated genes
DMRs: Differentially methylated regions
GAPDH: Glyceraldehyde 3-phosphate dehydrogenase
GO: Gene Ontology
ICD-10: International Classification of Diseases, Tenth Edition
IMA: Illumina Methylation Analyzer
MTHFR: 5,10-Methylenetetrahydrofolate reductase gene
MTRR: Methionine synthase reductase
NTDs: Neural tube defects
PCR: Polymerase chain reaction
PMSF: Phenylmethanesulfonyl fluoride
PVDF: Polyvinylidene difluoride
SB: Spina bifida
TRIM: Tripartite motif
