# Supplementary material for: TRIM4 is associated with neural tube defects based on genome-wide DNA methylation analysis
Source: Clin Epigenetics. 2019 Feb 1;11:17. doi: 10.1186/s13148-018-0603-z (PMC6359777; doi:10.1186/s13148-018-0603-z)
Supplement: Supplementary file 1 — Table S1. Primers for real-time PCR. (DOCX 18 kb) [file 13148_2018_603_MOESM1_ESM.docx]

Table 1. Primers for Real-time PCR

| Gene symbol | Primer sequence (5’-3’) | Annealing temperature (℃) |
| --- | --- | --- |
| MAP2K2 | Sense:TCAGCGGTCACGGGATGGATA | 60 |
|  | Antisense:TTAGGAGGTGGCTCGTTCACAATA |  |
| CALCOCO2 | Sense: GTTACTTTGCCCATTGACCTA | 60 |
|  | Antisense: ACCACACCATCCTCATCCACA |  |
| TRIM4 | Sense:CACACAGTGGAAGGATAAGATAA | 60 |
|  | Antisense: ATGAAGCGATAGTTTGATTGAG |  |
| GNAS | Sense:  CCTGCTACGAACGCTCCAAC | 60 |
|  | Antisense: TCCTGATCGCTCGGCACATA |  |
| TLR1 | Sense: TGAACCTCAAGCACTTGGACC | 60 |
|  | Antisense: CCCATAAGTCTCTCCTAAGACCA |  |
| HSPB7 | Sense: GTGGGGCAGGCAACATCAAG | 60 |
|  | Antisense: ACCGCAAACTCATAGGCGTCTC |  |
| EGFR | Sense:CCAAGGCACGAGTAACAAGC | 60 |
|  | Antisense:AGGGCAATGAGGACATAACC |  |
| MAGT1 | Sense: CCGATTTGCCTGTTCTCAC | 60 |
|  | Antisense: ACACCATCTCCTTCTTTCTTTG |  |
| SCT | Sense: GCGAGCAGGACGCAGAGAACAGC | 60 |
|  | Antisense: CAGGGGGCAGCCAGGGAGACCA |  |
| SMPD3 | Sense:CTCCTTCATACCCACCACCTACGA | 60 |
|  | Antisense:AAGAGAAAGCCGAGAAACGCAA |  |
| GAPDH | Sense: GCACCGTCAAGGCTGAGAAC | 60 |
|  | Antisense:TGGTGAAGACGCCAGTGGA |  |
