# Supplementary material for: TRIM4 is associated with neural tube defects based on genome-wide DNA methylation analysis
Source: Clin Epigenetics. 2019 Feb 1;11:17. doi: 10.1186/s13148-018-0603-z (PMC6359777; doi:10.1186/s13148-018-0603-z)
Supplement: Supplementary file 2 — Table S2. Primers for pyrosequencing. (DOCX 17 kb) [file 13148_2018_603_MOESM2_ESM.docx]

Table S2. Primers for pryosequencing.

| Gene symbol | Primer symbol | Primer sequence (5’-3’) |
| --- | --- | --- |
| TLR1 | BB150153-TLR1-F | AGGGTTTTTTTTGTATAAGATTAAATAGT |
|  | BB150153-TLR1-R-bio | ATATTTCCCAAACTACATCCAATTT |
|  | BB150153-TLR1-S | AGAATTGTTTTGTTTTGTTTTTTA |
| TRIM4-1 | BB150153-TRIM4-1-F-bio | GAGGGGGTTGGTTTAGGTTTA |
|  | BB150153-TRIM4-1-R | CTTACCCAAATCCTCTATAACTTTCC |
|  | BB150153-TRIM4-1-S | CTATAACTATTTAAAAAAACCACT |
| TRIM4-2 | BB150153-TRIM4-2-F | GTTTTTTTAGTTAGTTTGGTTAGGG |
|  | BB150153-TRIM4-2-R-bio | ACCTAAACTATTTCCAAAACC |
|  | BB150153-TRIM4-2-S | GTTAGTTTGGTTAGGGTT |
