# Supplementary material for: TRIM4 is associated with neural tube defects based on genome-wide DNA methylation analysis
Source: Clin Epigenetics. 2019 Feb 1;11:17. doi: 10.1186/s13148-018-0603-z (PMC6359777; doi:10.1186/s13148-018-0603-z)
Supplement: Supplementary file 3 — Table S3. Functions of genes with differential hypomethylation. (Note: yellow marker represents that there are more than ten genes in this GO). (DOCX 25 kb) [file 13148_2018_603_MOESM3_ESM.docx]

Table S3.Functions of genes with differential hypomethylation (Note: Yellow marker represent that there are more than ten genes in this GO.)

| GO_ID | Description | TYPE | Gene Ratio | p-value | Count | Top 8 genes in each GO |
| --- | --- | --- | --- | --- | --- | --- |
| GO:0034341 | response to interferon-gamma | biological_process | 7/131 | 0 | 7 | HLA-DRA HLA-C CCL3 HLA-DQB2 LGALS9 CALCOCO2 HLA-B |
| GO:0071346 | cellular response to interferon-gamma | biological_process | 6/131 | 0.0002 | 6 | HLA-DRA HLA-C CCL3 HLA-DQB2 LGALS9 HLA-B |
| GO:0060333 | interferon-gamma-mediated signaling pathway | biological_process | 4/131 | 0.001 | 4 | HLA-DQB2 HLA-DRA HLA-C HLA-B |
| GO:0009306 | protein secretion | biological_process | 7/131 | 0.0014 | 7 | CCL3 ALOX15B GNAS CD40 GOLPH3L LGALS9 TLR1 |
| GO:0032680 | regulation of tumor necrosis factor production | biological_process | 4/131 | 0.0015 | 4 | CCL3 ZBTB20 LGALS9 TLR1 |
| GO:0032675 | regulation of interleukin-6 production | biological_process | 4/131 | 0.0015 | 4 | ZBTB20 LGALS9 HLA-B TLR1 |
| GO:0032640 | tumor necrosis factor production | biological_process | 4/131 | 0.0016 | 4 | CCL3 ZBTB20 LGALS9 TLR1 |
| GO:0050708 | regulation of protein secretion | biological_process | 6/131 | 0.0016 | 6 | ALOX15B CCL3 CD40 GOLPH3L LGALS9 TLR1 |
| GO:0032635 | interleukin-6 production | biological_process | 4/131 | 0.0018 | 4 | ZBTB20 LGALS9 HLA-B TLR1 |
| GO:0009063 | cellular amino acid catabolic process | biological_process | 4/131 | 0.0041 | 4 | SLC25A21 GAD1 ATP2B2 HIBADH |
| GO:0002573 | myeloid leukocyte differentiation | biological_process | 5/131 | 0.0047 | 5 | PRDM16 GNAS CCL3 MMP9 PDE2A |
| GO:0050714 | positive regulation of protein secretion | biological_process | 4/131 | 0.0056 | 4 | ALOX15B CCL3 GOLPH3L LGALS9 |
| GO:0050707 | regulation of cytokine secretion | biological_process | 4/131 | 0.0067 | 4 | ALOX15B CCL3 LGALS9 TLR1 |
| GO:0043281 | regulation of cysteine-type endopeptidase activity involved in apoptotic process | biological_process | 5/131 | 0.0081 | 5 | BCL2L10 TNFAIP8 MMP9 LGALS9 MGMT |
| GO:0009617 | response to bacterium | biological_process | 9/131 | 0.0103 | 9 | CD160 CCL3 TNFAIP8 SRR LGALS9 TLR1 CHGA CD40 |
| GO:2000116 | regulation of cysteine-type endopeptidase activity | biological_process | 5/131 | 0.0106 | 5 | BCL2L10 TNFAIP8 MMP9 LGALS9 MGMT |
| GO:0019932 | second-messenger-mediated signaling | biological_process | 5/131 | 0.0111 | 5 | GNAS CDH13 CCL3 PDE2A ATP2B2 |
| GO:0001816 | cytokine production | biological_process | 10/131 | 0.0114 | 10 | ZBTB20 CCL3 TNFAIP8 LGALS9 TLR1 CHGA ZBP1 ALOX15B |
| GO:0050663 | cytokine secretion | biological_process | 4/131 | 0.012 | 4 | ALOX15B CCL3 LGALS9 TLR1 |
| GO:0007266 | Rho protein signal transduction | biological_process | 4/131 | 0.0123 | 4 | CDH13 ARHGEF10 ARHGEF3 ARHGEF4 |
| GO:0008217 | regulation of blood pressure | biological_process | 4/131 | 0.0153 | 4 | BDKRB2 TNNI3 CHGA ADAMTS16 |
| GO:0045087 | innate immune response | biological_process | 15/131 | 0.0161 | 15 | TRIM4 HLA-DQB2 LGALS9 TLR1 MAP2K2 CALCOCO2 ZBP1 HLA-C |
| GO:0001819 | positive regulation of cytokine production | biological_process | 6/131 | 0.0179 | 6 | ZBTB20 CCL3 ZBP1 ALOX15B CD40 LGALS9 |
| GO:0007565 | female pregnancy | biological_process | 4/131 | 0.02 | 4 | PNOC GNAS MMP9 LGALS9 |
| GO:0030099 | myeloid cell differentiation | biological_process | 6/131 | 0.0215 | 6 | PRDM16 CCL3 TRIM10 GNAS MMP9 PDE2A |
| GO:0050900 | leukocyte migration | biological_process | 6/131 | 0.0248 | 6 | CHGA SLC7A11 CCL3 SPNS2 MMP9 LGALS9 |
| GO:0006869 | lipid transport | biological_process | 5/131 | 0.0259 | 5 | ESYT3 SPNS2 ANO4 BDKRB2 PITPNC1 |
| GO:0006874 | cellular calcium ion homeostasis | biological_process | 6/131 | 0.0259 | 6 | TNNI3 CDH23 CCL3 ATP2B2 BDKRB2 CD40 |
| GO:0048002 | antigen processing and presentation of peptide antigen | biological_process | 4/131 | 0.0263 | 4 | HLA-DRA HLA-C HLA-DQB2 HLA-B |
| GO:0007160 | cell-matrix adhesion | biological_process | 4/131 | 0.0278 | 4 | TNXB CDH13 UTRN NID2 |
| GO:0002520 | immune system development | biological_process | 11/131 | 0.0296 | 11 | MAD1L1 PRDM16 CCL3 TRIM10 MMP9 LGALS9 SPNS2 GNAS |
| GO:0055074 | calcium ion homeostasis | biological_process | 6/131 | 0.0306 | 6 | TNNI3 CDH23 CCL3 ATP2B2 BDKRB2 CD40 |
| GO:0050776 | regulation of immune response | biological_process | 13/131 | 0.0308 | 13 | FCGR2A CD160 HLA-DQB2 LGALS9 TLR1 MAP2K2 GRIN2D SPNS2 |
| GO:0051321 | meiotic cell cycle | biological_process | 4/131 | 0.0312 | 4 | SYCP1 TUBGCP3 PIWIL1 SGOL2 |
| GO:0007173 | epidermal growth factor receptor signaling pathway | biological_process | 6/131 | 0.0322 | 6 | MAP2K2 REPS2 CDH13 GRIN2D MMP9 ADAM12 |
| GO:0051223 | regulation of protein transport | biological_process | 7/131 | 0.0336 | 7 | CCL3 ALOX15B CD40 GOLPH3L LGALS9 PDE2A TLR1 |
| GO:0002366 | leukocyte activation involved in immune response | biological_process | 4/131 | 0.0343 | 4 | CHGA CCL3 CD40 LGALS9 |
| GO:0002521 | leukocyte differentiation | biological_process | 7/131 | 0.0352 | 7 | PRDM16 CCL3 GNAS MMP9 LGALS9 PDE2A HLA-B |
| GO:0038127 | ERBB signaling pathway | biological_process | 6/131 | 0.0353 | 6 | MAP2K2 REPS2 CDH13 GRIN2D MMP9 ADAM12 |
| GO:0002263 | cell activation involved in immune response | biological_process | 4/131 | 0.0355 | 4 | CHGA CCL3 CD40 LGALS9 |
| GO:0052548 | regulation of endopeptidase activity | biological_process | 6/131 | 0.0362 | 6 | TFPI2 BCL2L10 TNFAIP8 MMP9 LGALS9 MGMT |
| GO:0001817 | regulation of cytokine production | biological_process | 8/131 | 0.0403 | 8 | ZBTB20 CCL3 LGALS9 TLR1 ZBP1 ALOX15B CD40 HLA-B |
| GO:0010876 | lipid localization | biological_process | 5/131 | 0.0428 | 5 | ESYT3 SPNS2 ANO4 BDKRB2 PITPNC1 |
| GO:0006955 | immune response | biological_process | 19/131 | 0.0438 | 19 | CD160 TRIM4 HLA-DQB2 LGALS9 TLR1 MAP2K2 CALCOCO2 ZBP1 |
| GO:0006952 | defense response | biological_process | 20/131 | 0.0444 | 20 | CD160 TRIM4 HLA-DQB2 LGALS9 TLR1 MAP2K2 CALCOCO2 ZBP1 |
| GO:0010033 | response to organic substance | biological_process | 28/131 | 0.0468 | 28 | SYP CDH13 RNF111 ARHGEF3 GNAS SLC7A11 ATP2B2 HTRA4 |
| GO:0052547 | regulation of peptidase activity | biological_process | 6/131 | 0.04690 | 6 | TFPI2 BCL2L10 TNFAIP8 MMP9 LGALS9 MGMT |
| GO:0046395 | carboxylic acid catabolic process | biological_process | 4/131 | 0.04751 | 4 | SLC25A21 GAD1 HIBADH ATP2B2 |
| GO:0002443 | leukocyte mediated immunity | biological_process | 5/131 | 0.04778 | 5 | CHGA CCL3 CD40 LGALS9 HLA-B |
| GO:0002237 | response to molecule of bacterial origin | biological_process | 5/131 | 0.04974 | 5 | CCL3 SRR CD40 LGALS9 TLR1 |
