# Supplementary material for: TRIM4 is associated with neural tube defects based on genome-wide DNA methylation analysis
Source: Clin Epigenetics. 2019 Feb 1;11:17. doi: 10.1186/s13148-018-0603-z (PMC6359777; doi:10.1186/s13148-018-0603-z)
Supplement: Supplementary file 4 — Table S4. Functions of genes with differential hypermethylation. (Note: yellow marker represents that there are more than ten genes in this GO). (DOCX 27 kb) [file 13148_2018_603_MOESM4_ESM.docx]

Table S4. Functions of genes with differential hypermethylation (Note: Yellow marker represent that there are more than ten genes in this GO.)

| **GO_ID** | **Description** | **TYPE** | **Gene Ratio** | **p-value** | **Count** | **Top 8 genes in each GO** |
| --- | --- | --- | --- | --- | --- | --- |
| GO:0031295 | T cell costimulation | biological_process | 4/78 | 0.0000 | 4 | HLA-DQB1 HLA-DRB5 HLA-DQA2 HLA-DQA1 |
| GO:0060333 | interferon-gamma-mediated signaling pathway | biological_process | 4/78 | 0.0001 | 4 | HLA-DQB1 HLA-DRB5 HLA-DQA2 HLA-DQA1 |
| GO:0019886 | antigen processing and presentation of exogenous peptide antigen via MHC class II | biological_process | 4/78 | 0.0001 | 4 | HLA-DQB1 HLA-DRB5 HLA-DQA2 HLA-DQA1 |
| GO:0002495 | antigen processing and presentation of peptide antigen via MHC class II | biological_process | 4/78 | 0.0001 | 4 | HLA-DQB1 HLA-DRB5 HLA-DQA2 HLA-DQA1 |
| GO:0002504 | antigen processing and presentation of peptide or polysaccharide antigen via MHC class II | biological_process | 4/78 | 0.0002 | 4 | HLA-DQB1 HLA-DRB5 HLA-DQA2 HLA-DQA1 |
| GO:0006664 | glycolipid metabolic process | biological_process | 4/78 | 0.0003 | 4 | PIGQ SERAC1 SMPD3 PIGG |
| GO:0006643 | membrane lipid metabolic process | biological_process | 5/78 | 0.0004 | 5 | PIGQ SMPD3 PIGG SERAC1 LASS3 |
| GO:0071346 | cellular response to interferon-gamma | biological_process | 4/78 | 0.0007 | 4 | HLA-DRB5 HLA-DQB1 HLA-DQA2 HLA-DQA1 |
| GO:0046488 | phosphatidylinositol metabolic process | biological_process | 4/78 | 0.0008 | 4 | PIGQ PIGG SERAC1 PIP5K1C |
| GO:0050851 | antigen receptor-mediated signaling pathway | biological_process | 5/78 | 0.0009 | 5 | PLCL2 HLA-DRB5 HLA-DQB1 HLA-DQA2 HLA-DQA1 |
| GO:0006629 | lipid metabolic process | biological_process | 14/78 | 0.0010 | 14 | SERAC1 PIGG FADS3 SULT1A2 LMF1 PIGQ ATP5A1 SMPD3 |
| GO:0019882 | antigen processing and presentation | biological_process | 5/78 | 0.0012 | 5 | HLA-DRB5 MICB HLA-DQB1 HLA-DQA2 HLA-DQA1 |
| GO:0034341 | response to interferon-gamma | biological_process | 4/78 | 0.0013 | 4 | HLA-DRB5 HLA-DQB1 HLA-DQA2 HLA-DQA1 |
| GO:0002429 | immune response-activating cell surface receptor signaling pathway | biological_process | 6/78 | 0.0015 | 6 | PLCL2 MICB HLA-DRB5 HLA-DQB1 HLA-DQA2 HLA-DQA1 |
| GO:0050852 | T cell receptor signaling pathway | biological_process | 4/78 | 0.0016 | 4 | HLA-DRB5 HLA-DQB1 HLA-DQA2 HLA-DQA1 |
| GO:0044255 | cellular lipid metabolic process | biological_process | 11/78 | 0.0018 | 11 | PIGQ SERAC1 LASS3 EHHADH SMPD3 PIGG PRKAG2 CYP2E1 |
| GO:0042176 | regulation of protein catabolic process | biological_process | 5/78 | 0.0021 | 5 | DACT1 BAT3 RNF144B EGFR RNF217 |
| GO:0006839 | mitochondrial transport | biological_process | 4/78 | 0.0025 | 4 | SLC25A37 TOMM34 PRKAG2 ATP5A1 |
| GO:0048002 | antigen processing and presentation of peptide antigen | biological_process | 4/78 | 0.0031 | 4 | HLA-DRB5 HLA-DQB1 HLA-DQA2 HLA-DQA1 |
| GO:0050870 | positive regulation of T cell activation | biological_process | 4/78 | 0.0032 | 4 | HLA-DRB5 HLA-DQB1 HLA-DQA2 HLA-DQA1 |
| GO:0008610 | lipid biosynthetic process | biological_process | 8/78 | 0.0035 | 8 | PIGQ SERAC1 LASS3 SMPD3 PIGG PRKAG2 PIP5K1C FADS3 |
| GO:0042886 | amide transport | biological_process | 5/78 | 0.0037 | 5 | SMPD3 SCT SLC38A7 GLUL HLA-DRB5 |
| GO:0002768 | immune response-regulating cell surface receptor signaling pathway | biological_process | 7/78 | 0.0041 | 7 | HLA-DQB1 PLCL2 MICB HLA-DRB5 EGFR HLA-DQA2 HLA-DQA1 |
| GO:0006644 | phospholipid metabolic process | biological_process | 5/78 | 0.0061 | 5 | PIGQ SMPD3 PIGG SERAC1 PIP5K1C |
| GO:0008654 | phospholipid biosynthetic process | biological_process | 4/78 | 0.0067 | 4 | PIGQ PIGG SERAC1 PIP5K1C |
| GO:0006082 | organic acid metabolic process | biological_process | 11/78 | 0.0069 | 11 | SLC35B3 GLUL WARS2 FADS3 SULT1A2 EGFLAM EHHADH PRKAG2 |
| GO:0051649 | establishment of localization in cell | biological_process | 19/78 | 0.0080 | 19 | TUBA1B SERAC1 SYNE1 SLC25A37 HLA-DRB5 EGFR SMPD3 SCT |
| GO:0006164 | purine nucleotide biosynthetic process | biological_process | 4/78 | 0.0084 | 4 | PRKAG2 ATP5A1 SCT SLC35B3 |
| GO:0006486 | protein glycosylation | biological_process | 5/78 | 0.0091 | 5 | GALNT9 MAGT1 MGAT5B DPY19L1 LMF1 |
| GO:0043413 | macromolecule glycosylation | biological_process | 5/78 | 0.0091 | 5 | GALNT9 MAGT1 MGAT5B DPY19L1 LMF1 |
| GO:0051641 | cellular localization | biological_process | 21/78 | 0.0093 | 21 | TUBA1B BAT3 SERAC1 SYNE1 SLC25A37 HLA-DRB5 ASPSCR1 IPO5 |
| GO:0009100 | glycoprotein metabolic process | biological_process | 6/78 | 0.0095 | 6 | GALNT9 EGFLAM MAGT1 MGAT5B DPY19L1 LMF1 |
| GO:0050890 | cognition | biological_process | 4/78 | 0.0096 | 4 | EGFR SCT SGK1 MAGT1 |
| GO:0051050 | positive regulation of transport | biological_process | 8/78 | 0.0098 | 8 | DACT1 IPO5 GLUL SGK1 TRPV3 SCT HLA-DRB5 EGFR |
| GO:0046390 | ribose phosphate biosynthetic process | biological_process | 4/78 | 0.0099 | 4 | PRKAG2 ATP5A1 SCT SLC35B3 |
| GO:0070085 | glycosylation | biological_process | 5/78 | 0.0100 | 5 | GALNT9 MAGT1 MGAT5B DPY19L1 LMF1 |
| GO:0030072 | peptide hormone secretion | biological_process | 4/78 | 0.0101 | 4 | SMPD3 SCT GLUL HLA-DRB5 |
| GO:0006650 | glycerophospholipid metabolic process | biological_process | 4/78 | 0.0104 | 4 | PIGQ PIGG SERAC1 PIP5K1C |
| GO:0002757 | immune response-activating signal transduction | biological_process | 6/78 | 0.0106 | 6 | PLCL2 MICB HLA-DRB5 HLA-DQB1 HLA-DQA2 HLA-DQA1 |
| GO:0002790 | peptide secretion | biological_process | 4/78 | 0.0116 | 4 | SMPD3 SCT GLUL HLA-DRB5 |
| GO:0002683 | negative regulation of immune system process | biological_process | 4/78 | 0.0118 | 4 | PLCL2 MICB HLA-DRB5 SERPINB9 |
| GO:1901990 | regulation of mitotic cell cycle phase transition | biological_process | 4/78 | 0.0141 | 4 | DACT1 MEPCE KNTC1 EGFR |
| GO:0050863 | regulation of T cell activation | biological_process | 4/78 | 0.0147 | 4 | HLA-DRB5 HLA-DQB1 HLA-DQA2 HLA-DQA1 |
| GO:0015833 | peptide transport | biological_process | 4/78 | 0.0147 | 4 | SMPD3 SCT GLUL HLA-DRB5 |
| GO:0008202 | steroid metabolic process | biological_process | 4/78 | 0.0147 | 4 | PRKAG2 CYP2E1 SULT1A2 LMF1 |
| GO:0002764 | immune response-regulating signaling pathway | biological_process | 7/78 | 0.0157 | 7 | HLA-DQB1 PLCL2 MICB HLA-DRB5 EGFR HLA-DQA2 HLA-DQA1 |
| GO:0050867 | positive regulation of cell activation | biological_process | 4/78 | 0.0188 | 4 | HLA-DRB5 HLA-DQB1 HLA-DQA2 HLA-DQA1 |
| GO:0048568 | embryonic organ development | biological_process | 5/78 | 0.0190 | 5 | FBN2 TPO SCT EGFR SLITRK6 |
| GO:1901987 | regulation of cell cycle phase transition | biological_process | 4/78 | 0.0190 | 4 | DACT1 MEPCE KNTC1 EGFR |
| GO:0046879 | hormone secretion | biological_process | 4/78 | 0.0193 | 4 | SMPD3 SCT GLUL HLA-DRB5 |
| GO:0009101 | glycoprotein biosynthetic process | biological_process | 5/78 | 0.0216 | 5 | GALNT9 MAGT1 MGAT5B DPY19L1 LMF1 |
| GO:0009914 | hormone transport | biological_process | 4/78 | 0.0217 | 4 | SMPD3 SCT GLUL HLA-DRB5 |
| GO:0042110 | T cell activation | biological_process | 5/78 | 0.0227 | 5 | MICB HLA-DRB5 HLA-DQB1 HLA-DQA2 HLA-DQA1 |
| GO:0009165 | nucleotide biosynthetic process | biological_process | 4/78 | 0.0245 | 4 | PRKAG2 SCT SLC35B3 ATP5A1 |
| GO:0034504 | protein localization to nucleus | biological_process | 4/78 | 0.0257 | 4 | DACT1 IPO5 EGFR SYNE1 |
| GO:0033365 | protein localization to organelle | biological_process | 7/78 | 0.0260 | 7 | TOMM34 DACT1 BAT3 IPO5 ZFAND2B SYNE1 EGFR |
| GO:0071345 | cellular response to cytokine stimulus | biological_process | 7/78 | 0.0276 | 7 | TUBA1B HLA-DQB1 SMPD3 HLA-DRB5 IL1F10 HLA-DQA2 HLA-DQA1 |
| GO:0010033 | response to organic substance | biological_process | 19/78 | 0.0285 | 19 | TUBA1B HSPB7 HLA-DRB5 HLA-DQA1 TBL1Y IPO5 MICB PRKAG2 |
| GO:0019221 | cytokine-mediated signaling pathway | biological_process | 6/78 | 0.0292 | 6 | HLA-DQB1 SMPD3 HLA-DRB5 IL1F10 HLA-DQA2 HLA-DQA1 |
| GO:0043161 | proteasome-mediated ubiquitin-dependent protein catabolic process | biological_process | 4/78 | 0.0310 | 4 | TBL1Y BAT3 RNF144B RNF217 |
| GO:0010817 | regulation of hormone levels | biological_process | 5/78 | 0.0316 | 5 | SMPD3 TPO SCT GLUL HLA-DRB5 |
| GO:0008104 | protein localization | biological_process | 15/78 | 0.0333 | 15 | BAT3 SERAC1 SYNE1 HLA-DRB5 ASPSCR1 IPO5 ZFAND2B EGFR |
| GO:0046649 | lymphocyte activation | biological_process | 6/78 | 0.0355 | 6 | HLA-DQB1 PLCL2 MICB HLA-DRB5 HLA-DQA2 HLA-DQA1 |
| GO:0050778 | positive regulation of immune response | biological_process | 6/78 | 0.0364 | 6 | HLA-DQB1 PLCL2 MICB HLA-DRB5 HLA-DQA2 HLA-DQA1 |
| GO:0006631 | fatty acid metabolic process | biological_process | 4/78 | 0.0365 | 4 | EHHADH PRKAG2 CYP2E1 FADS3 |
| GO:0006810 | transport | biological_process | 27/78 | 0.0370 | 27 | TUBA1B SLC35B3 ASPSCR1 IPO5 ZFAND2B ATP5A1 PRKAG2 SCT |
| GO:0005975 | carbohydrate metabolic process | biological_process | 8/78 | 0.0386 | 8 | PIGQ MAGT1 SLC35B3 MGAT5B DPY19L1 PRKAG2 GALNT9 LMF1 |
| GO:0010498 | proteasomal protein catabolic process | biological_process | 4/78 | 0.0413 | 4 | TBL1Y BAT3 RNF144B RNF217 |
| GO:0055085 | transmembrane transport | biological_process | 10/78 | 0.0444 | 10 | MAGT1 SLC35B3 SGK1 SLC25A37 TRPV3 ADD3 ZFAND2B ATP5A1 |
| GO:0009790 | embryo development | biological_process | 8/78 | 0.0484 | 8 | FBN2 DACT1 BAT3 ATP5A1 TPO SCT EGFR SLITRK6 |
| GO:0043687 | post-translational protein modification | biological_process | 4/78 | 0.0494 | 4 | PIGQ PIGG GALNT9 MAGT1 |
| GO:0006812 | cation transport | biological_process | 8/78 | 0.0494 | 8 | MAGT1 ATP5A1 SGK1 SLC25A37 TRPV3 PRKAG2 SLC38A7 ATP2A1 |
