# Supplementary material for: TRIM4 is associated with neural tube defects based on genome-wide DNA methylation analysis
Source: Clin Epigenetics. 2019 Feb 1;11:17. doi: 10.1186/s13148-018-0603-z (PMC6359777; doi:10.1186/s13148-018-0603-z)
Supplement: Supplementary file 5 — S1. CpG site information for pyrosequencing in TRIM4, TLR1, and MAP2K2. Pyrosequencing was performed for identifying the methylation level of different transcription regulatory regions in TRIM4, TLR1, and MAP2K2 in a larger sample size. However, the pyrosequencing test was only conducted in TRIM4 and TLR1 due to the low primer score of MAP2K2. The cg20606062, cg09654046, cg22087659, cg02016764, and cg24748945 mentioned below are the probe number of the differential methylation site in the microarray analysis. (DOCX 29 kb) [file 13148_2018_603_MOESM5_ESM.docx]

File S1. Pyrosequencing was performed for identifying the methylation level of different transcription regulatory regions in TRIM4, TLR1 and MAP2K2 in a larger sample size. However, the pyrosequencing test were only conducted in TRIM4 and TLR1 due to the low primer score of MAP2K2. The cg20606062, cg09654046, cg22087659, cg02016764 and cg24748945 mentioned below are the probe number of the differential methylation site in the microarray analysis.

1. Assessment for the cg20606062, cg09654046 and cg22087659 sites of TRIM4 along with other CpG sites around them (the sequences with underline were the target sequences, the sites marked with box were cg20606062, cg09654046 and cg22087659, respectively).

cg20606062 and cg09654046:

>hg19_dna range=chr7:99516779-99517779 5'pad=500 3'pad=500 strand=+ repeatMasking=none

CACGGGGCCCAGGCGCCGGCGCTGCGTCTTCTCAGTCAGCCTGGCCAGGGCCCAGTTGGGTCGCAGCGCGGCGGGCGCCGATGGGTGCCGACATTCGGGGCAGGGGAACGGGCCGCCGCCCGGCGCCCAGTTGCGGTGCAGGCAGCCGCGGCAGAAGTTGTGGCCGCACTCGATGGACACCGGGTCCTGGAAATAGTCCAGGCAGATGGGGCAGGTCAACTCCTCCTGGATGTCCTCAGCTTCCATGCTGCTTCCCTGCCGCGGAGACGGAGTCCGACGTGAGGCGCGGGAGAGGCCAGCAAGCTGCGAGCGGCCGCGGGGAGGCCAGACGACTTCCGAACCGCCGTCACCGCCTCACGTAAAAGGGTACAACGCAGTTTCTCTTCCGGGGTTCAGGACCCAAAAAGAACGCACGGACGTTGCCCGGCCTGAGCGCGCAAGACCGGAAGCTGCTGGGTATCCGCGCCGGAACCGCGAGGGGGTTGGTTCAGGCCTAGGCGCGGGGCAGGACGGGACCGGTGAGTGGCTCCTCCAAACAGCTATAGAGACCCAGAAATGCCTGTGGAAAGCTACAGAGGACTTGGGCAAGGCGTTAGAGCCGGACGTTGTGGTCCCTGGGGGAGCCAACCGGGGAGGTGCCTATGATCTTTCTTTCCAAGTTTGAGGGTTCAGGTCTGTGTTCGCCTGCTGGTTAGACCGATTTAAGTTTTAGAGTCCCAGGAGTTCTGGGCGAGCTGCAGCCTAGGGGCCTGGAAGGCCAGGAAGACTTGAAGGATCTGTGTTGGTAGGCCTGGAGTTTGGCTAACGTGGGGATGATTTGGATTCTGAGATGCAGATACATTGCCTTTTTGGTATCCAATTTGAAAAGTAGCAACCATTTTGCTATCCCAAAGAACATCCATGGAGGCTTTCAACAAATAATATGCTCACCTGTTTCAGGGAATTATCTATATCCCTTAAGTGGATTTTCGATCTTGTGTTTCTGAGGTGCTTTGCTTTTT

| **Primer Set 2** | | | **Score: 89 Quality: High** | | |  |
| --- | --- | --- | --- | --- | --- | --- |
| **Primer** | **Id** | **Sequence** | **Nt** | **Tm, ºC** | **%GC** |  |
| 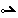PCR | F2 | GAGGGGGTTGGTTTAGGTTTA | 21 | 59.0 | 47.6 |  |
| 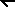PCR | R2 | CTTACCCAAATCCTCTATAACTTTCC | 26 | 59.5 | 38.5 |  |
| 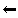Sequencing | S2 | CTATAACTATTTAAAAAAACCACT | 24 | 44.7 | 20.8 |  |
| Target Polymorphisms | Position15, Position16, Position17, Position18 | | | | | |
| Sequence to Analyze | CACCRATCCC RTCCTACCCC RCRCCTAAAC CTAAACCAAC CCCCTC | | | | |  |

cg22087659 :

>hg19_dna range=chr7:99516345-99517345 5'pad=500 3'pad=500 strand=+ repeatMasking=none

AGCGGCACACACCCTCTCAGTATGTTTTTCTTTATTATGAGCTCCCCAAGGGCCAGTTTATTGAAAATTCTGCACAATGATTGCTGAGGTTTGCACCTCCCCTCCTCTCATTCCAGTTTTAACCCTAGCTCTGAAATTCGTGTTTTGCTTAACTTCTTTGAGTTTCAGGGGCTTTTCATGCTTCCAGTAGGAATGACAGCCACTTCGTGGCTCTTTTAGGAGCATTAAGTAAACTTTATCGGGCAACACTGACAGCCCCGTCATAGTCACCGCGACGGCCAGCTCACCCGGTAGCTCTCGAAGGCCTCGTCGATGGGTGCCATGGCGTGAGTCTGGTGCTCCTGGGACTCCCTGCACACCAGGCACACTGGCCGCTGGTCGTCCTCGCAGAAGAGCCGCAGCGGCTCCCAGTGGCGGCCGCACAGGCCCGGGGGCACGGGGCCCAGGCGCCGGCGCTGCGTCTTCTCAGTCAGCCTGGCCAGGGCCCAGTTGGGTCGCAGCGCGGCGGGCGCCGATGGGTGCCGACATTCGGGGCAGGGGAACGGGCCGCCGCCCGGCGCCCAGTTGCGGTGCAGGCAGCCGCGGCAGAAGTTGTGGCCGCACTCGATGGACACCGGGTCCTGGAAATAGTCCAGGCAGATGGGGCAGGTCAACTCCTCCTGGATGTCCTCAGCTTCCATGCTGCTTCCCTGCCGCGGAGACGGAGTCCGACGTGAGGCGCGGGAGAGGCCAGCAAGCTGCGAGCGGCCGCGGGGAGGCCAGACGACTTCCGAACCGCCGTCACCGCCTCACGTAAAAGGGTACAACGCAGTTTCTCTTCCGGGGTTCAGGACCCAAAAAGAACGCACGGACGTTGCCCGGCCTGAGCGCGCAAGACCGGAAGCTGCTGGGTATCCGCGCCGGAACCGCGAGGGGGTTGGTTCAGGCCTAGGCGCGGGGCAGGACGGGACCGGTGAGTGGCTCCTCCAAACAGCTATAGAGACCCAGAAATGCCTGTGGAA

| **Primer Set 1** | | | **Score: 78 Quality: Medium** | | |  |
| --- | --- | --- | --- | --- | --- | --- |
| **Primer** | **Id** | **Sequence** | **Nt** | **Tm, ºC** | **%GC** |  |
| 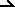PCR | F1 | GTTTTTTTAGTTAGTTTGGTTAGGG | 25 | 57.2 | 32.0 |  |
| 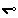PCR | R1 | ACCTAAACTATTTCCAAAACC | 21 | 54.8 | 33.3 |  |
| 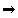Sequencing | S1 | GTTAGTTTGGTTAGGGTT | 18 | 46.4 | 38.9 |  |
| Target Polymorphisms | Position14, Position15, Position16, Position17, Position18, Position19 | | | | | |
| Sequence to Analyze | TAGTTGGGTY GTAGYGYGGY GGGYGTYGAT GGGTGTYGAT ATT | | | | |  |

2. Assessment for the cg02016764 site of TLR1 along with other CpG sites around it (the sequences with underline are the target sequences, the site marked with box was cg02016764).

>hg19_dna range=chr4:38804732-38806732 5'pad=1000 3'pad=1000 strand=+ repeatMasking=none

CTAAGCAGCCAGGACAAGGCGGACAGTCTGAGAGAACTACTGTAAGTAAGCTGCTAATGAGAGCTGTTGCTGAATAAAATCACCTTTCACCTGCCTACGGCCCCCTGAGTGTTCTTTCAGCTATCTGCTCATCCACCCCCTCCCCTCAGACCTCAGTATGGGCTGGAACCTGGCCCTGGGCGTGACACTCTCTCTCTTACTCTAAAACTTGCCTCGGTCTCTCACTCTGCCTTATGCCCCTCACACAAATTCTTTCCACTGAGGAGGCAAGAATTAAGTTTCTACGACCCATATGGATTCACCACTGCTAACGCAAGCATTTAATGAAAGCATAGCTTCAGTTTTATGTCGAGCTAAATTAATTGACTCAAATATCTTCTATTTGCCTAACCTCCATCTTGCCCTTTCATTTAAAGTCATTAATTTTAAGTACTCAGTTTATGAAACCATTCTTATTTCTATAATTAAGAAATCAAAATGTTATACACAGGAATAACTGATTTTTAATACTTCCAGGATCTTTGAATTTTATATACCAAACTTGCTAAATCCATGAAAATCAATGTCTCAGTTTCCTAGACCAAAGAATTTTTTGATCAACGTCTTAATGACCATGACTGAGCCTCATCTATTTTAGAACCTTTAAACTCTTTTACTATCCAAAAAATAAGCTGAGCAGAAAAGGATGCTTTAATTTGAAAGTGTAAAATGCCTCTAGGTGAGCTTCTGTGAATTTTCATTATTATGATAGGTTTAGCCTCACATTATATGATACAATGAATCAATACATTTAATTTGTCTTCTTTTGGAGACTTTGGAGGCATCAAATCAAGGCTTTGACTGGTCTTAATGGACAATACCCATACAATCTATCTGGCCCATTAAGTTTAGTCCTGTAAATATTTACTAAGGGCCTTCTCTGCACAAGACCAAATAGCTATTAACCAAAGGTTAAAAGAATCTGAGACAGTTCAAGAACTGTTCTGCTTTGCTTTCTATGCGGTTTTGTGAACTCTTGCTTTGTTATAAAACAAATTCACTTTCAAGTCTTTTGACCAAATTGGATGTAGCCTGGGAAACATTTTCAAAACCTGCAATTCTACCTCACGTCTAGCTCCCCAGAACAGTGTTAGTGGCCTGAGAAACAGAAGGACTAGCTAGTGGGAAGGCCCCAGAGAGAAAAACTGAAACAAACCTGGCCACAAAAACAGAAGAGCTGAACAGCAGCATTGCCTCCGGGAGTAACTGACTTATATCTCATTTTCTTTAACACTGAGAAGAGAATACAAATGGCGGGATTAGGTGAAGAGAAATGCTGTCAAAATCAACAACTAGCAGCTGCTCTGGAAGAAAACCAGAAGACGTGTGTGACCCAAATATTCATTTCCCAACTCAAAAATATTTCCTATAAGCAAATCACACACGAGGTGTTATTCCCAGAGGAGTGTGGGGACTTTCTCTGATTTAGGACTCAGAACTGATACACAAATGCCAGTACAAACTACACAGTAACTATATACATATCAGAAATATAACCCACTTAGAAAAACACCCAGGACTTACACAGATTTTTGAGACTTCTGTACTTAATAACTAGCTATACAATTATTTTCTTTTACAAAAGAAAAGAATGTGTTGCTTACCAAGACAACCTGCTTGTCTGTTCCATTTGGCAGTCTGTAAGAAATTCAAGCACTTCCTTGAATTTATTTCACTGTTAACCACATTTGGTGTTGAAAACAAAACAAAACAAAAAGGCTGTTTTATGCCACCTACCGAATTCCACTGGAATTACATTACATAGTTAAGAATAAACTTATTTTCTCTTATTCATATGTAGCATAGTCTGTGGGTTACCTGATAGGCCTGTATTAAAATAGAATCACGTTTCTATGGTTCCAAATTTCACTACAGTCAGTTTACTATTGTCTACACTGATGAAGTAAATGAGGTAATGTTAATAATCCACAGATCATAATTGGCTGAATTCAAAATGCTTAC

| **Primer Set 1** | | | **Score: 81 Quality: Medium** | | |
| --- | --- | --- | --- | --- | --- |
| **Primer** | **Id** | **Sequence** | **Nt** | **Tm, ºC** | **%GC** |
| 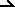PCR | F1 | AGGGTTTTTTTTGTATAAGATTAAATAGT | 29 | 57.7 | 20.7 |
| 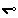PCR | R1 | ATATTTCCCAAACTACATCCAATTT | 25 | 57.9 | 28.0 |
| 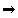Sequencing | S1 | AGAATTGTTTTGTTTTGTTTTTTA | 24 | 45.3 | 16.7 |
| Target Polymorphisms | Position1 | | | | |
| Sequence to Analyze | TGYGGTTTTG TGAATTTTTG TTTTGTTAT | | | | |

1. Assessment for the cg24748945 site of MAP2K2 along with other CpG sites around it (the sequences with underline are the target sequences, the sites marked with box was cg24748945).

>hg19_dna range=chr19:4122852-4124852 5'pad=1000 3'pad=1000 strand=+ repeatMasking=none

CCTATCTCATTATTGAGGGACCCTTACCCCGTCTTTTCCTACCAGGGACCCTACCCCATCTCAGTCTCATTACTCAGGGACCCCTCACCCCATCCTCTCCCCTCTGGAGCCCCCTTGCCCAGATTTCCTCCTCAGGGGCTGCCTGACCTCCCCACACCCCATCCTCTCCTCTCAGGAATGCCCTGCACCCCGATTTCCCTACTCAGGGTCTCCCCTCACCCTCATCTCATGACTACGGCAACCTTTACTCCGTCCTTTCCTCTTAGGAACTCTTCACCCTCATTTCACTACTCAAGGACCCCACACTGTGTTCTCTCCTCTTAGAGATACCCCTCACCCCGATCTCACTGCTTGGGGAGCCTCATCCTGTTCTCTCCTTGGGGACACCCCTCATTCCGATTTTCCTACTGAGGGACTCCCCTCACCCCAGCTTCACTTTTCAGAAGCCCCACACCCCATCTTCTGCACTGCAGGGACCTCCCCTCACTCAACCTCGCTGCTCAGGGACCCCTGTCCCCATCCTCTCCTCTTAGAGACATCCTTCACCCCAACTTCACTACTCAGAGACTCCCGTTATCCCAATTCCACTCTTCAGGAACCCCACACCCCATCTTTTCCACCACAAGGACCTCCCCTCGCCCAATCTCACTGCTCAAAGACCCCCCTGCCCCGTGCACCCTTCGCCCCGTCCTCCGAGGGCCCCCTGCCCCGTCCTCCCCCGAGGGCCCCCTGCCCCGTCCTCCCCCGAGGGTCCCCTGCCCTGTCCTCCCCTCGAGAACCCCCTGCCCCGTGCACCACTCACCCCGTCCTCCCCCGTGACCCCCCTGCCTCGTGCACTCCTCGCGAACCCCCGTCCCCTCGCCCCGTCCTTCCCCGAGGGCTCCCTGCCCCGTGCACCCCAAGCCTCCGGCTGACCCCTGCCCACTCACTCGGAGGCGCCCTCGCTGGTAGGGGATGGGCCCTCGGCGATGGTAGGGTTGATGGTGAGCGCCGGCAGCACCGGCTTCCTCCGGGCCAGCATCGGGGCTCCGCGGGCCGGCGGCGGCGGCGCCTCTAGCCGGGGCCCATAGGGGGCGGGCCGGGAGCGGTCGGCGCCTACGCGAGCCCGGGGCTGCGGCCGCGGCCCAGGCCGGCGTCGGGGCGGCCGAGGGCGGGCGGCGCTGCGGGCCTGGGCCGAGGGTAGCCGAGGGGCGCTGGGGCTGAGGCGAGCGAGCCGCTACCGCTGCCGAGGCCCGAAGAAGGCTGACGCCGCAGCCCGAGTCCGAGAGGCAGGGGGAGGGGAGGGGCGGCCACAAGATCGCGGACCGGCTTCTCGCGATAACGGGATCGGGAGCCGCGATGGACCCCACCCCCAGCCGCCGCGCCTGCGCAGCAGCACAAGGCCGCCTTTCGGAGGGGAAAGGGGCGGAGACCGACGCGAGGCGGTGCCGGGACCGGGCGCCCTCGCTTGCCTTACCACGGCGCGTGTGCCCAAGCGCTTGGGGCATGAGGCGCGGGAGCAGGGGAGGAGACGGAGGGATGAAGGCTGGGGTGATGGTCGTGCACAGGTGGGCAGGGTAGCCAGACTCCTGAGAGCCTGTGGTGCCCCAGGCTGGGGCTGAGAGAACCCGTAAGACCCCAGAACAGTGGACGAGACCCACCCAGAGTCCATTTAATCTTTTTGGCAAGCGTGGACACACGCCCCTAGCCCCCACCGCCTTAGAGTGTCAGTTACTCCCCCTCCTTCATCAGAACAAGGCCACTCTAAAACATTTTTAAAATTTATTTTAATTGACAAATAAAATTGCATATATTACTATGCGCATGTTGAAACATTTTTATTATAAAATACACATAACATAAAACTTGTCATTTTAACCATTTCTAAATGCACAGCTCAGCGGCATTAAGCAACCACCACCACCACCATCTCCAGAATTTTCTCATCTTCCGAAACTGAAACACTGTCCCCTATGAAACCTCACTCCCTATCCCCCTCCCCAGCCCCTGGAACCCAACGTC

| **Primer Set 1** | | | **Score: 65 Quality: Medium** | | |  |
| --- | --- | --- | --- | --- | --- | --- |
| **Primer** | **Id** | **Sequence** | **Nt** | **Tm, ºC** | **%GC** |  |
| 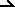PCR | F1 | TGGTAGGGGATGGGTTTT | 18 | 59.7 | 50.0 |  |
| 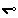PCR | R1 | ACCCCCCCCCTATAAACCC | 19 | 56.4 | 63.2 |  |
| 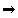Sequencing | S1 | AGGGTTGATGGTGAG | 15 | 48.5 | 53.3 |  |
| Target Polymorphisms | Position10, Position11, Position12, Position13, Position14, Position15, Position16 | | | | | |
| Sequence to Analyze | YGTYGGTAGT ATYGGTTTTT TTYGGGTTAG TATYGGGGTT TYGYGGGTYG GYGGYGG | | | | |  |

**Note: the score is too low to be recommended.**
