# Supplementary material for: TRIM4 is associated with neural tube defects based on genome-wide DNA methylation analysis
Source: Clin Epigenetics. 2019 Feb 1;11:17. doi: 10.1186/s13148-018-0603-z (PMC6359777; doi:10.1186/s13148-018-0603-z)
Supplement: Supplementary file 6 — S2. The sequence information of the regions targeted for pyrosequencing in TRIM4. (Notes: the underline represents the pyrosequencing fragment of TRIM4 in promoter and exon 1. Yellow font represents the primers of Sanger sequencing. Red font represents the detected CpG site. Blue font represents the first exon of TRIM4. Green font represents the missense mutation. Purple highlight represents the cg09654046, cg20606062, and cg22087659, respectively). (DOCX 14 kb) [file 13148_2018_603_MOESM6_ESM.docx]

File S2. The sequence and methylated region of *TRIM4* gene (Notes: The underline represent the prosequencing fragment of *TRIM4* in promoter and exon 1. Yellow font represent the primers of Sanger sequencing. Red font represent the detected CpG site. Blue font represent the first exon of TRIM4. Green font represent the missense mutation. Purple highlight represent the cg09654046, cg20606062 and cg22087659, respectively.)

1 tcataggcac ctccccggtt ggctccccca gggaccacaa cgtccggctc taacgccttg

61 cccaagtcct ctgtagcttt ccacaggcat ttctgggtct ctatagctgt ttggaggagc

121 cactcaccgg tcccgtcctg ccccgcgcct aggcctgaac caaccccctc gcggttccgg

181 cgcggatacc cagcagcttc cggtcttgcg cgctcaggcc gggcaacgtc cgtgcgttct

241 ttttgggtcc tgaaccccgg aagagaaact gcgttgtacc cttttacgtg aggcggtgac

301 ggcggttcgg aagtcgtctg gcctccccgc ggccgctcgc agcttgctgg cctctcccgc

361 gcctcacgtc ggactccgtc tccgcggcag ggaagcagca tggaagctga ggacatccag

421 gaggagttga cctgccccat ctgcctggac tatttccagg acccggtgtc catcgagtgc

481 ggccacaact tctgccgcgg ctgcctgcac cgcaactggg cgccgggcgg cggcccgttc

541 ccctgccccg aatgtcggca cccatcggcg cccgccgcgc tgcgacccaa ctgggccctg

601 gccaggctga ctgagaagac gcagcgccgg cgcctgggcc cc**g**tgccccc gggcctgtgc

661 ggccgccact gggagccgct gcggctcttc tgcgaggacg accagcggcc agtgtgcctg

721 gtgtgcaggg agtcccagga gcaccagact cacgccatgg cacccatcga cgaggccttc

781 gagagctacc gggtgagctg gccgtcgcgg tgactatgac ggggctgtca gtgttgcccg

841 ataaagttta cttaatgctc ctaaaagagc cacgaagtgg ctgtcattcc tactggaagc

901 atgaaaagcc cctgaaactc aaagaagtta agcaaaacac gaatttcaga gctagggtta
